# Supplementary material for: Phosphine-Functionalized Core-Crosslinked Micelles and Nanogels with an Anionic Poly(styrenesulfonate) Shell: Synthesis, Rhodium(I) Coordination and Aqueous Biphasic Hydrogenation Catalysis
Source: Polymers (Basel). 2022 Nov 15;14(22):4937. doi: 10.3390/polym14224937 (PMC9697678; doi:10.3390/polym14224937)
Supplement: Supplementary file 1 [file polymers-14-04937-s001.zip › polymers-2009927-supplementary.pdf]

SUPPORTING INFORMATION

Index

**(a) TPP-functionalized CCMs and NGs with P(SS^-Na^+) homopolymer blocks in the hydrophilic shell**		S4
Table S 1.	List of all polymers with P(SS^-Na^+) shell synthesized in this study and reference to their characterization.	S4
Figure S 1.	^{1}H NMR monitoring of the St/DPPS copolymerization for the chain extension of R_0-(SS^-Na^+)$_{140}$-*b*-St_{50}-SC(S)S*n*Pr to yield R_0-(SS^-Na^+)$_{140}$-*b*-St_{50}-*b*-(St_{1-y}-*co*-$DPPS_y$)$_{300}$-SC(S)S*n*Pr.	S5
Figure S 2.	^{1}H NMR monitoring of the St/DPPS copolymerization for the chain extension of R_0-(SS^-Na^+)$_{140}$-SC(S)S*n*Pr to yield R_0-(SS^-Na^+)$_{140}$-*b*-(St_{1-y}-*co*-$DPPS_y$)$_{300}$-SC(S)S*n*Pr.	S6
Figure S 3.	DLS and TEM characterization of the amphiphilic di(tri)-block copolymers, R_0-(SS^-Na^+)$_{140}$-*b*-St_x-*b*-(St_{1-y}-*co*-$DPPS_y$)$_{300}$-SC(S)S*n*Pr (x,y = 50,0.20; 0,0.05; 0,0.10; 0,0.20).	S7
Figure S 4.	^{1}H and ^{31}P NMR spectra of (a) R_0-(SS^-Na^+)$_{140}$-*b*-($St_{0.8}$-*co*-$DPPS_{0.2}$)$_{300}$-SC(S)S*n*Pr and (b) R_0-(SS^-Na^+)$_{140}$-*b*-St_{50}-*b*-($St_{0.8}$-*co*-$DPPS_{0.2}$)$_{300}$-SC(S)S*n*Pr latexes in D_2O/$CDCl_3$ (3:1 v/v).	S8
Figure S 5.	^{1}H NMR monitoring of the St/DEGDMA copolymerization for the crosslinking of R_0-(SS^-Na^+)$_{140}$-*b*-St_{50}-*b*-(St_{1-y}-*co*-$DPPS_y$)$_{300}$-SC(S)S*n*Pr to yield the CCMs R_0-(SS^-Na^+)$_{140}$-*b*-St_{50}-*b*-(St_{1-y}-*co*-$DPPS_y$)$_{300}$-*b*-($St_{0.9}$-*co*-$DEGDMA_{0.1}$)$_{150}$-SC(S)S*n*Pr (y = 0.05, 0.1, 0.2).	S9
Figure S 6.	Comparison of unfiltered and filtered DLS traces of the aqueous dispersions of the CCMs R_0-(SS^-Na^+)$_{140}$-*b*-St_{50}-*b*-($St_{0.95}$-*co*-$DPPS_{0.05}$)$_{300}$-*b*-($St_{0.90}$-*co*-$DEGDMA_{0.10}$)$_{150}$-SC(S)S*n*Pr.	S10
Figure S 7.	Comparison of unfiltered and filtered DLS traces of the aqueous dispersions of the CCMs R_0-(SS^-Na^+)$_{140}$-*b*-St_{50}-*b*-($St_{0.90}$-*co*-$DPPS_{0.10}$)$_{300}$-*b*-($St_{0.90}$-*co*-$DEGDMA_{0.10}$)$_{150}$-SC(S)S*n*Pr.	S11
Figure S 8.	Comparison of unfiltered and filtered DLS traces of the aqueous dispersions of the CCMs R_0-(SS^-Na^+)$_{140}$-*b*-St_{50}-*b*-($St_{0.80}$-*co*-$DPPS_{0.20}$)$_{300}$-*b*-($St_{0.90}$-*co*-$DEGDMA_{0.10}$)$_{150}$-SC(S)S*n*Pr.	S12
Figure S 9.	^{1}H and ^{31}P NMR spectra of R_0-(SS^-Na^+)$_{140}$-*b*-St_{50}-*b*-(St_{1-y}-*co*-$DPPS_y$)$_{300}$-*b*-($St_{0.9}$-*co*-$DEGDMA_{0.1}$)$_{150}$-SC(S)S*n*Pr latexes (y = 0.05, 0.1, 0.2) in D_2O/$CDCl_3$ (3:1 v/v).	S13
Figure S 10.	^{1}H NMR monitoring of the DEGDMA polymerization for the crosslinking of R_0-(SS^-Na^+)$_{140}$-*b*-(St_{1-y}-*co*-$DPPS_y$)$_{300}$-SC(S)S*n*Pr to yield the CCMs R_0-	S14

	(SS$^-$Na$^+$)$_{140}$-*b*-(St$_{1-y}$-*co*-DPPS$_y$)$_{300}$-*b*-DEGDMA$_{15}$-SC(S)S*n*Pr (y = 0.05, 0.1, 0.2, 0.25).	
Figure S 11.	^{1}H and ^{31}P NMR spectra of R$_0$-(SS$^-$Na$^+$)$_{140}$-*b*-(St$_{1-y}$-*co*-DPPS$_y$)-*b*-DEGDMA$_{15}$-SC(S)S*n*Pr latexes (y = 0.05, 0.1, 0.2, 0.25) in D$_2$O/CDCl$_3$ (3:1 v/v).	S15
Figure S 12.	Comparison of unfiltered and filtered DLS traces of the aqueous dispersions of the CCMs R$_0$-(SS$^-$Na$^+$)$_{140}$-*b*-(St$_{0.95}$-*co*-DPPS$_{0.05}$)$_{300}$-*b*-DEGDMA$_{15}$-SC(S)S*n*Pr.	S16
Figure S 13.	Comparison of unfiltered and filtered DLS traces of the aqueous dispersions of the CCMs R$_0$-(SS$^-$Na$^+$)$_{140}$-*b*-(St$_{0.90}$-*co*-DPPS$_{0.10}$)$_{300}$-*b*-DEGDMA$_{15}$-SC(S)S*n*Pr.	S17
Figure S 14.	Comparison of unfiltered and filtered DLS traces of the aqueous dispersions of the CCMs R$_0$-(SS$^-$Na$^+$)$_{140}$-*b*-(St$_{0.80}$-*co*-DPPS$_{0.20}$)$_{300}$-*b*-DEGDMA$_{15}$-SC(S)S*n*Pr.	S18
Figure S 15.	Comparison of unfiltered and filtered DLS traces of the aqueous dispersions of the CCMs R$_0$-(SS$^-$Na$^+$)$_{140}$-*b*-(St$_{0.80}$-*co*-DPPS$_{0.20}$)$_{300}$-*b*-DEGDMA$_{15}$-SC(S)S*n*Pr.	S19
Figure S 16.	NMR data for the synthesis of the NGs R$_0$-(SS$^-$Na$^+$)$_{140}$-*b*-St$_x$-*b*-(St$_y$-*co*-DPPS$_z$-*co*-DEGDMA$_{15}$)-SC(S)S*n*Pr (x,y,z = 0,285,15; 50,425,30).	S20
Figure S 17.	Comparison of unfiltered and filtered DLS traces of the aqueous dispersions of the NG R$_0$-(SS$^-$Na$^+$)$_{140}$-*b*-(St$_{285}$-*co*-DPPS$_{15}$-*co*-DEGDMA$_{15}$)-SC(S)S*n*Pr.	S21
Figure S 18.	Comparison of unfiltered and filtered DLS traces of the aqueous dispersions of the NG R$_0$-(SS$^-$Na$^+$)$_{140}$-*b*-St$_{50}$-*b*-(St$_{425}$-*co*-DPPS$_{30}$-*co*-DEGDMA$_{15}$)-SC(S)S*n*Pr.	S22
Figure S 19.	Photos of the CCM and NG polymer latexes after treatment with a toluene solution of [RhCl(COD)]$_2$ (P/Rh = 1:1).	S22
**(b) ^{1}H NMR investigations of the Rh-sulfonate interaction**		S23
Figure S 20.	^{1}H NMR spectrum of the [RhCl(COD)]$_2$/NaO$_3$SC$_6$H$_4$-4-CH$_3$ interaction (Rh/sulfonate = ca. 1:5) in DMSO-*d*$_6$/D$_2$O (4:1 v/v).	S23
Figure S 21.	^{1}H NMR spectrum of the [RhCl(COD)(PPh$_3$)]/NaO$_3$SC$_6$H$_4$-4-CH$_3$ interaction (Rh/sulfonate = ca. 1:5) in DMSO-*d*$_6$/D$_2$O (4:1 v/v).	S24
Figure S 22.	^{1}H NMR spectrum of the [RhCl(COD)]$_2$/R$_0$-(SS$^-$Na$^+$)$_{140}$-SC(S)S*n*Pr interaction (Rh/sulfonate = ca. 1:5) in DMSO-*d*$_6$/D$_2$O (6:1 v/v).	S25
Figure S 23.	^{1}H NMR spectrum of the [RhCl(COD)(PPh$_3$)]/R$_0$-(SS$^-$Na$^+$)$_{140}$-SC(S)S*n*Pr interaction (Rh/sulfonate = ca. 1:5) in DMSO-*d*$_6$/D$_2$O (6:1 v/v).	S26
**(c) TPP-functionalized CCMs and NGs with P(SS$^-$Na$^+$-*co*-PEOMA) copolymer blocks in the hydrophilic shell**		S27
Table S 2.	List of all polymers with P(SS$^-$Na$^+$-*co*-PEOMA) shell synthesized in this study and reference to their characterization.	S27
Figure S 24.	^{1}H NMR monitoring of the SS$^-$Na$^+$/PEOMA copolymerization for the synthesis of the R$_0$-[(SS$^-$Na$^+$)$_{0.2}$-*co*-PEOMA$_{0.8}$]$_{140}$-SC(S)S*n*Pr macroRAFT agents.	S28
Figure S 25.	^{1}H NMR and SEC monitoring of the SS$^-$Na$^+$/PEOMA RAFT copolymerization.	S29

Figure S 26. DLS (left, unfiltered) and TEM (right) characterization of R$_0$-[(SS$^-$Na$^+$)$_{0.2}$-*co*-PEOMA$_{0.8}$]$_x$-SC(S)S*n*Pr (x = 50, 140).	S30
Figure S 27. ^{1}H NMR spectra of R$_0$-[(SS$^-$Na$^+$)$_{0.2}$-*co*-PEOMA$_{0.8}$]$_x$-SC(S)S*n*Pr.	S31
Figure S 28. DLS and TEM characterization of R$_0$-[(SS$^-$Na$^+$)$_{0.2}$-*co*-PEOMA$_{0.8}$]$_x$-*b*-St$_{50}$-SC(S)S*n*Pr (x = 50, 140).	S32
Figure S 29. NMR data for the synthesis of the R$_0$-[(SS$^-$Na$^+$)$_{0.2}$-*co*-PEOMA$_{0.8}$]$_x$-*b*-(St$_{0.9}$-*co*-DPPS$_{0.1}$)$_{300}$-SC(S)S*n*Pr diblock copolymer (x = 50, 140).	S33
Figure S 30. DLS (left, unfiltered) and TEM (right) characterization of R$_0$-[(SS$^-$Na$^+$)$_{0.2}$-*co*-PEOMA$_{0.8}$]$_{50}$-*b*-(St$_{0.9}$-*co*-DPPS$_{0.1}$)$_{300}$-SC(S)S*n*Pr.	S33
Figure S 31. NMR data for the synthesis of the R$_0$-[(SS$^-$Na$^+$)$_{0.2}$-*co*-PEOMA$_{0.8}$]$_x$-*b*-(St$_{0.9}$-*co*-DPPS$_{0.1}$)$_{300}$-*b*-(St$_{0.9}$-*co*-DEGDMA$_{0.1}$)$_{150}$-SC(S)S*n*Pr CCMs (x = 50, 140).	S34
Figure S 32. NMR data for the synthesis of the R$_0$-[(SS$^-$Na$^+$)$_{0.2}$-*co*-PEOMA$_{0.8}$]$_{50}$-*b*-(St$_{0.9}$-*co*-DPPS$_{0.1}$)$_{300}$-*b*-DEGDMA$_{90}$-SC(S)S*n*Pr CCM.	S35
Figure S 33. NMR data for the synthesis of the R$_0$-[(SS$^-$Na$^+$)$_{0.2}$-*co*-PEOMA$_{0.8}$]$_{50}$-*b*-St$_{50}$-*b*-(St$_{425}$-*co*-DPPS$_{30}$-*co*-DEGDMA$_{15}$)-SC(S)S*n*Pr NGs.	S36
Figure S 34. DLS and TEM characterization of the R$_0$-[(SS$^-$Na$^+$)$_{0.2}$-*co*-PEOMA$_{0.8}$]$_{50}$-*b*-St$_{50}$-*b*-(St$_{425}$-*co*-DPPS$_{30}$-*co*-DEGDMA$_{15}$)-SC(S)S*n*Pr NGs.	S36
Figure S 35. DLS characterization of (a) the R$_0$-(SS$^-$Na$^+$$_{0.2}$-*co*-PEOMA$_{0.8}$)$_{50}$-*b*-(St$_{0.9}$-*co*-DPPS$_{0.1}$)$_{300}$-SC(S)SPr diblock copolymer and (b) the corresponding R$_0$-(SS$^-$Na$^+$$_{0.2}$-*co*-PEOMA$_{0.8}$)$_{50}$-*b*-(St$_{0.9}$-*co*-DPPS$_{0.1}$)$_{300}$-*b*-(St$_{0.9}$-*co*-DEGDMA$_{0.1}$)$_{150}$-SC(S)SPr CCM in a THF-water 60:40 mixed solvent.	S37
Table S 3. Hydrogenation of neat styrene with the [RhCl(COD)(TPP)] precatalyst embedded in CCM nanoreactors	S38
Figure S 36. Recovered reaction mixtures after the recycling runs of Table 1 (entry 1).	S39
Figure S 37. Recovered reaction mixtures after the catalytic runs of Table 1. (a) Entry 6. (b) Entry 7.	S39
Figure S 38. DLS of the recovered organic phase from the recycling experiments of Table 1, measured after dilution in Et$_2$O without filtration.	S40

(a) TPP-functionalized CCMs and NGs with P(SS$^-$Na$^+$) homopolymer blocks in the hydrophilic shell

Table S 1. List of all polymers with a P(SS$^-$Na$^+$) shell synthesized in this study and reference to their characterization.

Formula [a]	SEC	^{1}H NMR	^{31}P NMR	DLS	TEM	ζ (mV)
**1. Diblock PSS$^-$Na$^+$-*co*-P(St-*co*-DPPS) micelles**						
R_0-(PSS$^-$Na$^+$)$_{140}$-*b*-St$_{50}$-*b*-(St$_{0.95}$-*co*-DPPS$_{0.05}$)$_{300}$-SC(S)S*n*Pr	/	Figure S 1		/	/	/
R_0-(PSS$^-$Na$^+$)$_{140}$-*b*-St$_{50}$-*b*-(St$_{0.9}$-*co*-DPPS$_{0.1}$)$_{300}$-SC(S)S*n*Pr	/	Figure S 1		/	/	/
R_0-(PSS$^-$Na$^+$)$_{140}$-*b*-St$_{50}$-*b*-(St$_{0.8}$-*co*-DPPS$_{0.2}$)$_{300}$-SC(S)S*n*Pr	/	Figure S 1<br>Figure S 4	Figure S 4	Figure S 3	Figure S 3	/
R_0-(PSS$^-$Na$^+$)$_{140}$-*b*-(St$_{0.95}$-*co*-DPPS$_{0.05}$)$_{300}$-SC(S)S*n*Pr	/	Figure S 2		Figure S 3	Figure S 3	-53.5±2.1
R_0-(PSS$^-$Na$^+$)$_{140}$-*b*-(St$_{0.9}$-*co*-DPPS$_{0.1}$)$_{300}$-SC(S)S*n*Pr	/	Figure S 2		Figure S 3	Figure S 3	-50.6±3.9
R_0-(PSS$^-$Na$^+$)$_{140}$-*b*-(St$_{0.8}$-*co*-DPPS$_{0.2}$)$_{300}$-SC(S)S*n*Pr	/	Figure S 2<br>Figure S 4	Figure S 4	Figure S 3	Figure S 3	-49.7±7.0
R_0-(PSS$^-$Na$^+$)$_{140}$-*b*-(St$_{0.75}$-*co*-DPPS$_{0.25}$)$_{300}$-SC(S)S*n*Pr	/	Figure S 2		/	/	
**2. CCMs with a PSS$^-$Na$^+$ shell and a mixed DEGDMA-*co*-St core**						
R_0-(PSS$^-$Na$^+$)$_{140}$-*b*-St$_{50}$-*b*-(St$_{0.95}$-*co*-DPPS$_{0.05}$)$_{300}$-*b*-(St$_{0,9}$-*co*-DEGDMA$_{0,1}$)$_{150}$-SC(S)S*n*Pr	/	Figure S 5<br>Figure S 9	Figure S 9	Figure 3<br>Figure S 6	Figure 3	-50.0±1.9
R_0-(PSS$^-$Na$^+$)$_{140}$-*b*-St$_{50}$-*b*-(St$_{0.9}$-*co*-DPPS$_{0.1}$)$_{300}$-*b*-(St$_{0,9}$-*co*-DEGDMA$_{0,1}$)$_{150}$-SC(S)S*n*Pr	/	Figure S 5<br>Figure S 9	Figure S 9	Figure 3<br>Figure S 7	Figure 3	-50.5±0.6
R_0-(PSS$^-$Na$^+$)$_{140}$-*b*-St$_{50}$-*b*-(St$_{0.8}$-*co*-DPPS$_{0.2}$)$_{300}$-*b*-(St$_{0,9}$-*co*-DEGDMA$_{0,1}$)$_{150}$-SC(S)S*n*Pr	/	Figure S 5<br>Figure S 9	Figure S 9	Figure 3<br>Figure S 8	Figure 3	-51.8±1.6
**3. CCMs with a PSS$^-$Na$^+$ shell and a neat DEGDMA core**						
R_0-(PSS$^-$Na$^+$)$_{140}$-*b*-(St$_{0.95}$-*co*-DPPS$_{0.05}$)$_{300}$-*b*-DEGDMA$_{15}$-SC(S)S*n*Pr	/	Figure S 10<br>Figure S 11	Figure S 11	Figure 4<br>Figure S 12	Figure 4	-51.6±1.2
R_0-(PSS$^-$Na$^+$)$_{140}$-*b*-(St$_{0.9}$-*co*-DPPS$_{0.1}$)$_{300}$-*b*-DEGDMA$_{15}$-SC(S)S*n*Pr	/	Figure S 10<br>Figure S 11	Figure S 11	Figure 4<br>Figure S 13	Figure 4	-54.0±2.3
R_0-(PSS$^-$Na$^+$)$_{140}$-*b*-(St$_{0.8}$-*co*-DPPS$_{0.2}$)$_{300}$-*b*-DEGDMA$_{15}$-SC(S)S*n*Pr	/	Figure S 10<br>Figure S 11	Figure S 11	Figure 4<br>Figure S 14	Figure 4	-69.8±2.3
R_0-(PSS$^-$Na$^+$)$_{140}$-*b*-(St$_{0.75}$-*co*-DPPS$_{0.25}$)$_{300}$-*b*-DEGDMA$_{15}$-SC(S)S*n*Pr	/	Figure S 10<br>Figure S 11	Figure S 11	Figure 5<br>Figure S 15	Figure 5	-49.7±6.8
**4. NGs with PSS$^-$Na$^+$ shell**						
R_0-(PSS$^-$Na$^+$)$_{140}$-*b*-(St$_{285}$-*co*-DPPS$_{15}$-*co*-DEGDMA$_{15}$)-SC(S)S*n*Pr	/	Figure S 16	Figure S 16	Figure 5	Figure 5<br>Figure S 17	-51.2±1.2
R_0-(PSS$^-$Na$^+$)$_{140}$-*b*-St$_{50}$-*b*-(St$_{425}$-*co*-DPPS$_{30}$-*co*-DEGDMA$_{15}$)-SC(S)S*n*Pr	/	Figure S 16	Figure S 16	Figure 5	Figure 5<br>Figure S 18	-52.6±2.2

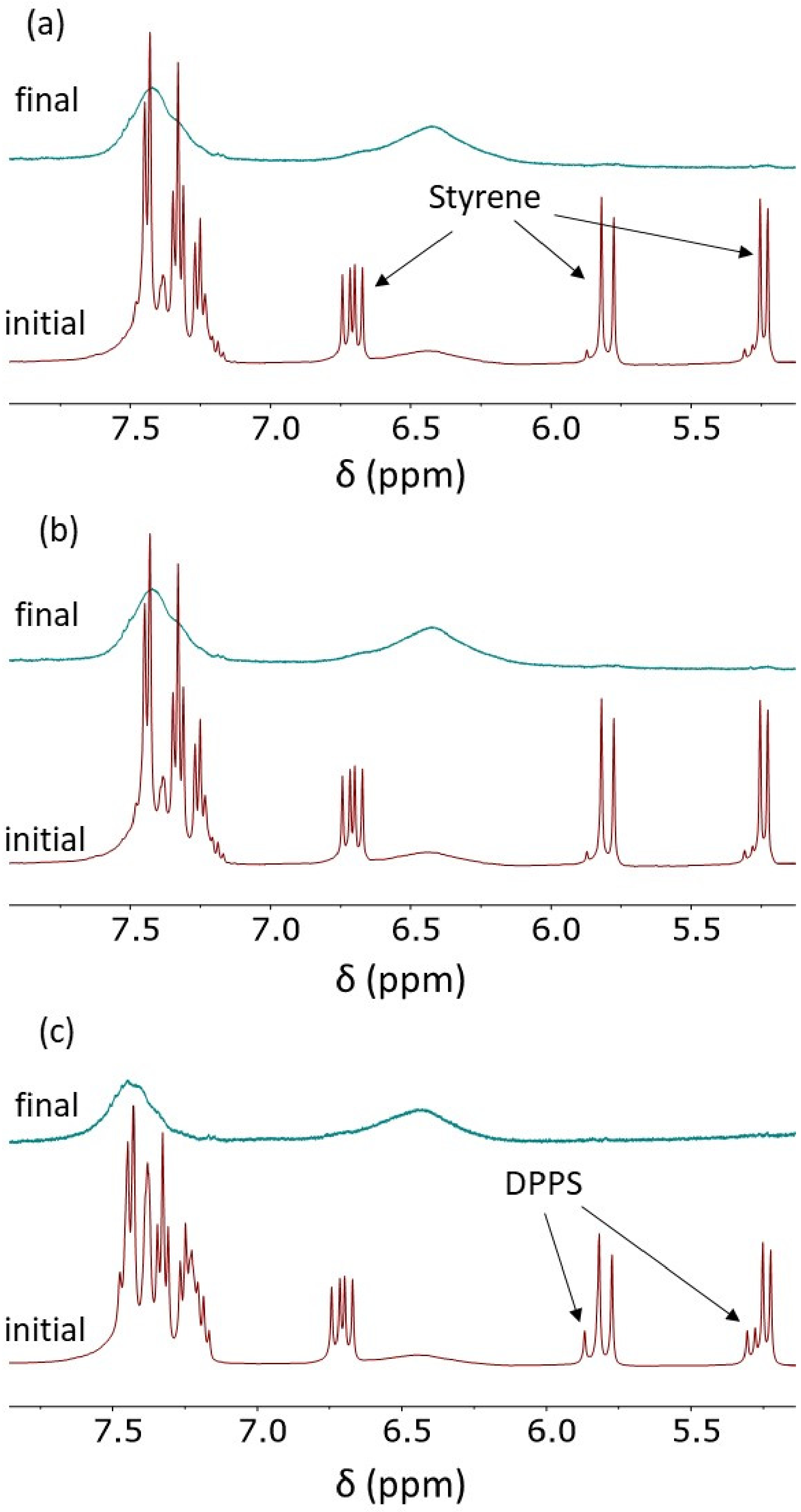

Figure S 1. ^{1}H NMR monitoring of the St/DPPS copolymerization for the chain extension of R_0-$(SS^-Na^+)_{140}$-*b*-St_{50}-SC(S)S*n*Pr to yield R_0-$(SS^-Na^+)_{140}$-*b*-St_{50}-*b*-$(St_{1-y}$-*co*-$DPPS_y)_{300}$-SC(S)S*n*Pr. (a) y = 0.05; (b) y = 0.10; (c) y = 0.20. All NMR samples were prepared by adding a drop of the reaction mixture directly to the DMSO-d_6 solvent in the NMR tube.

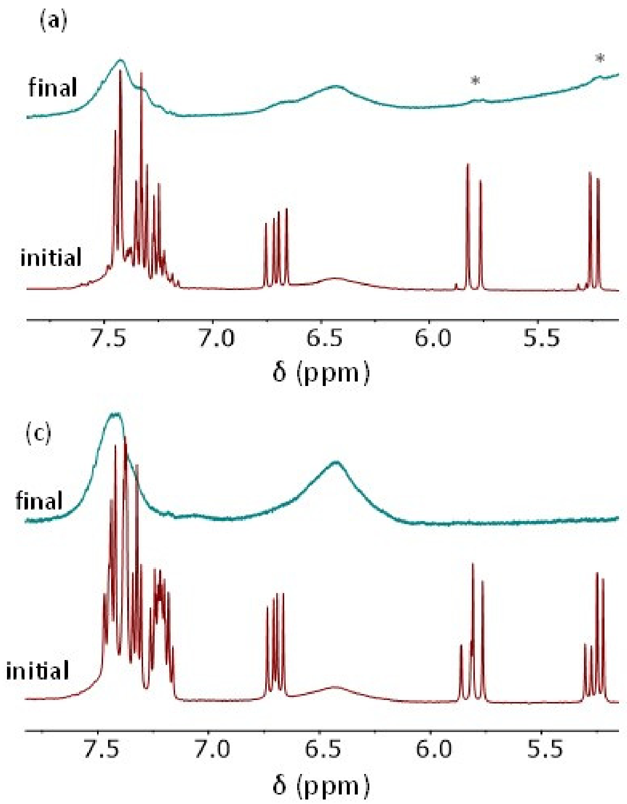

Figure S 2. ^{1}H NMR monitoring of the St/DPPS copolymerization for the chain extension of R_0-$(SS^{-}Na^{+})_{140}$-SC(S)S*n*Pr to yield R_0-$(SS^{-}Na^{+})_{140}$-*b*-$(St_{1-y}$-*co*-$DPPS_y)_{300}$-SC(S)S*n*Pr. (a) y = 0.05; (b) y = 0.10; (c) y = 0.20; (d) y = 0.25. The starred resonances correspond to residual styrene (< 1% by integration against the trioxane standard). All NMR samples were prepared by adding a drop of the reaction mixture directly to the DMSO-d_6 solvent in the NMR tube.

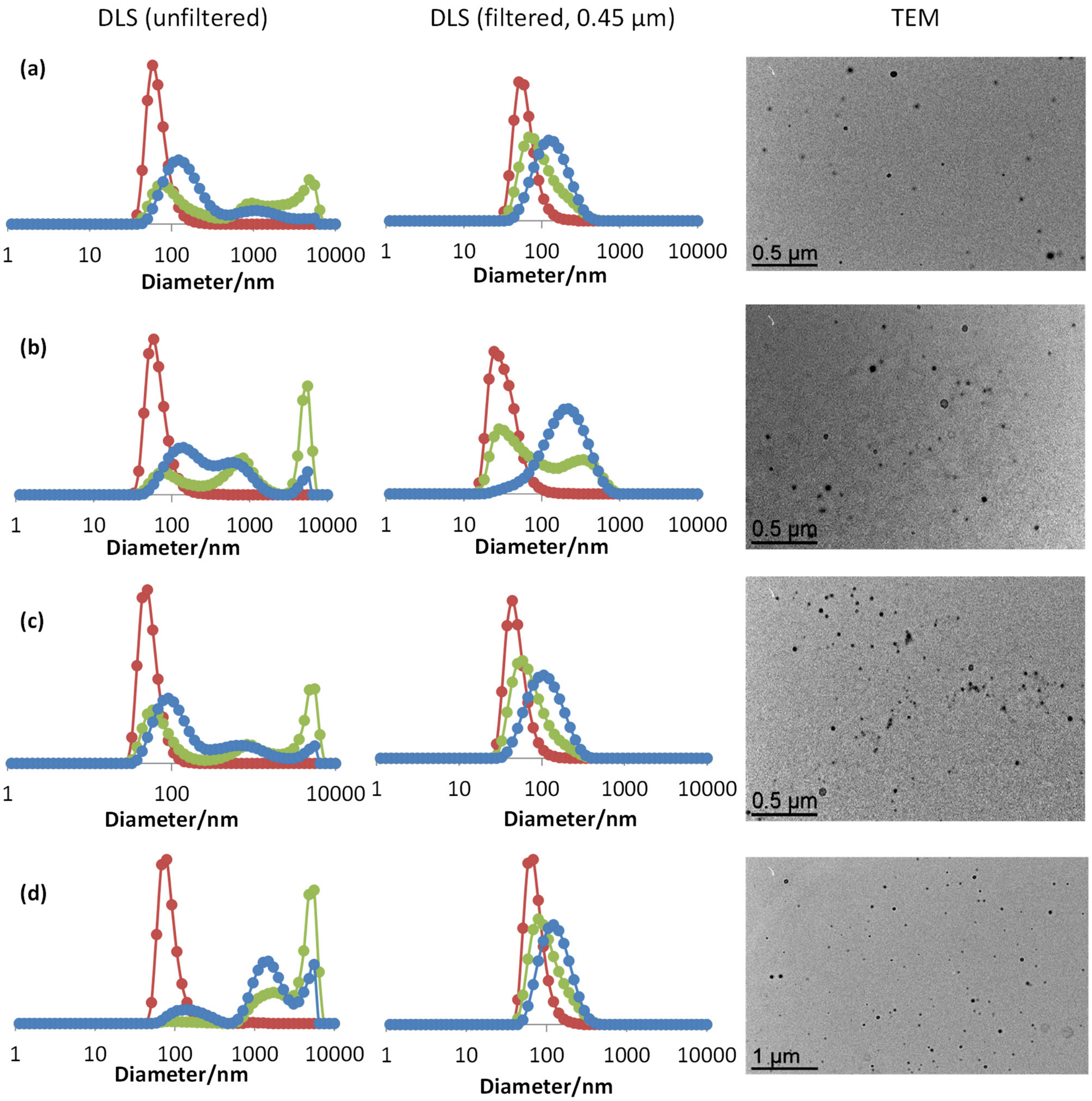

Figure S 3. DLS and TEM characterization of the amphiphilic di(tri)-block copolymers, R_0-$(SS^-Na^+)_{140}$-*b*-St_x-*b*-$(St_{1-y}$-*co*-$DPPS_y)_{300}$-SC(S)S*n*Pr. (a) x = 50, y = 0.20; (b) x = 0, y = 0.05; (c) x = 0, y = 0.10; (d) x = 0, y = 0.20. Color coding for the DLS size distributions: number (red), volume (green) and intensity (blue).

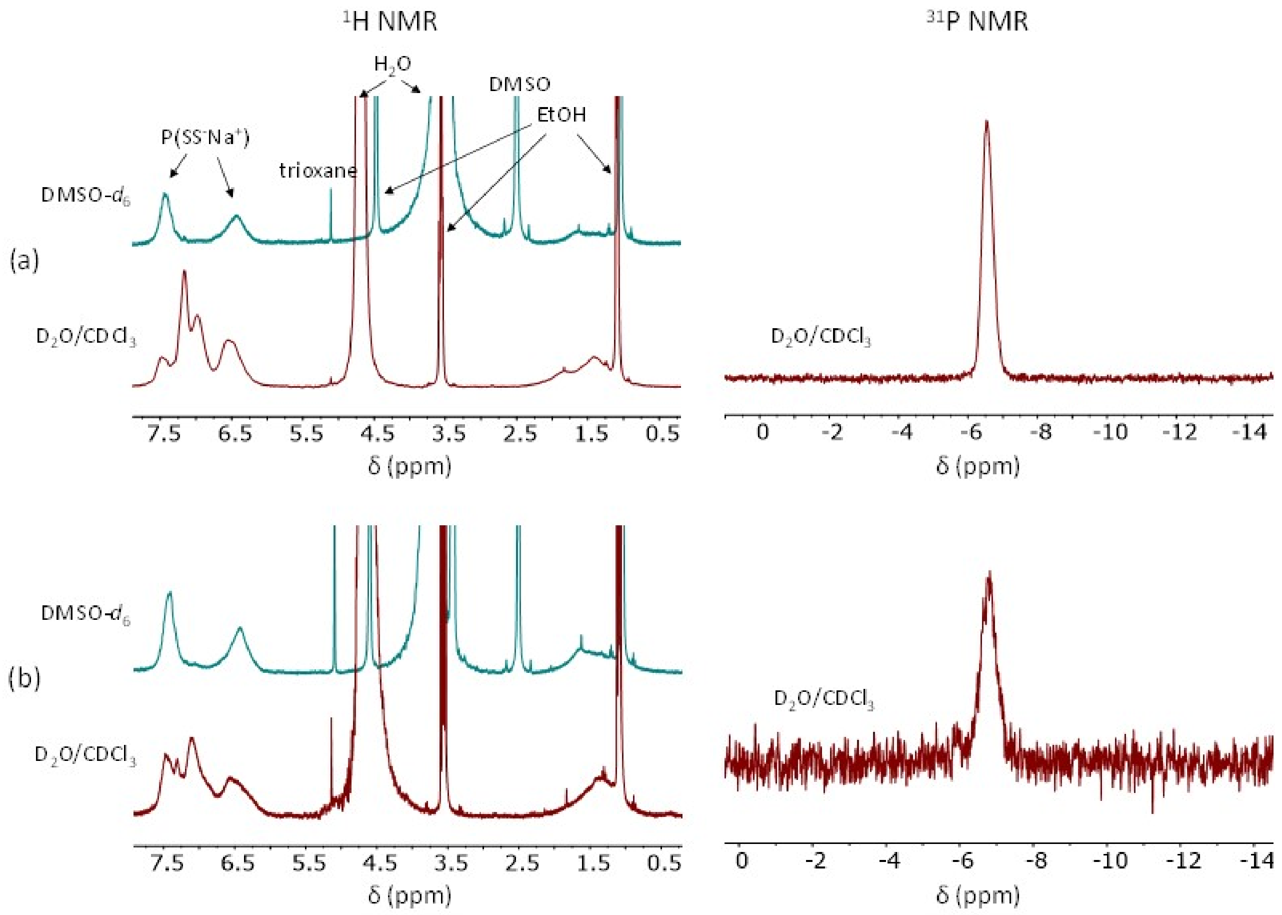

Figure S 4. ^{1}H and ^{31}P NMR spectra of (a) R_0-($SS^{-}Na^{+}$)$_{140}$-*b*-($St_{0.8}$-*co*-$DPPS_{0.2}$)$_{300}$-SC(S)S*n*Pr and (b) R_0-($SS^{-}Na^{+}$)$_{140}$-*b*-St_{50}-*b*-($St_{0.8}$-*co*-$DPPS_{0.2}$)$_{300}$-SC(S)S*n*Pr latexes in $D_2O/CDCl_3$ (3:1 v/v). The ^{1}H NMR spectra in DMSO-d_6 are also shown for comparison.

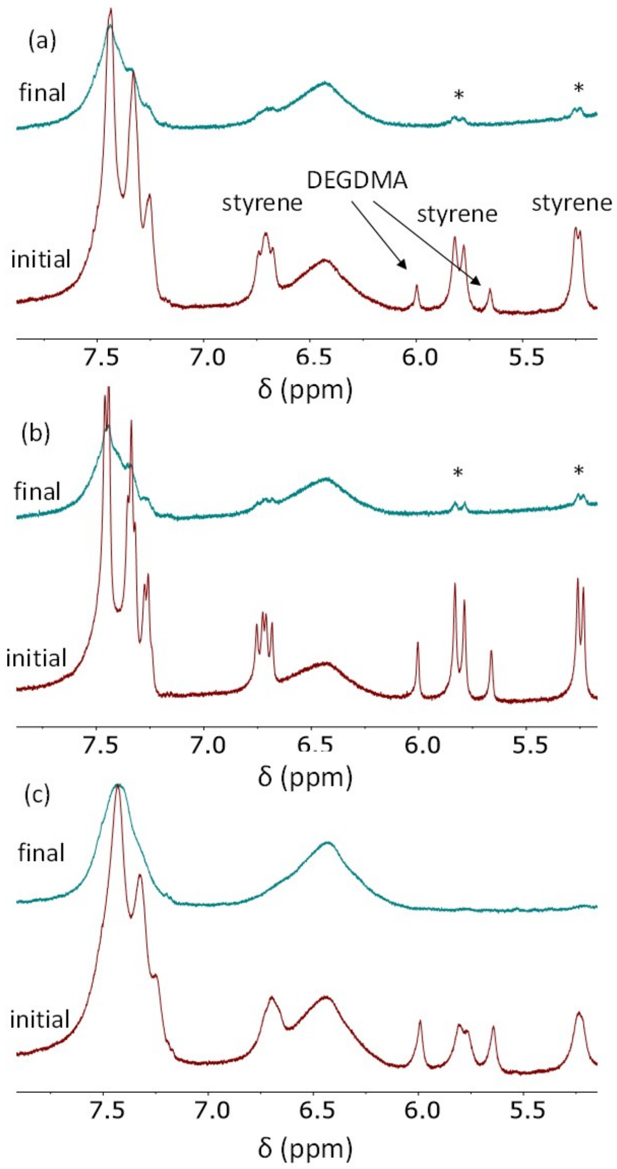

Figure S 5. ^{1}H NMR monitoring of the St/DEGDMA copolymerization for the crosslinking of R_0-$(SS^-Na^+)_{140}$-*b*-St_{50}-*b*-$(St_{1-y}$-*co*-$DPPS_y)_{300}$-SC(S)S*n*Pr to yield the CCMs R_0-$(SS^-Na^+)_{140}$-*b*-St_{50}-*b*-$(St_{1-y}$-*co*-$DPPS_y)_{300}$-*b*-$(St_{0.9}$-*co*-$DEGDMA_{0.1})_{150}$-SC(S)S*n*Pr. (a) y = 0.05; (b) y = 0.10; (c) y = 0.20. The starred resonances correspond to residual styrene (< 1% by integration against the trioxane standard). All NMR samples were prepared by adding a drop of the reaction mixture directly to the DMSO-d_6 solvent in the NMR tube.

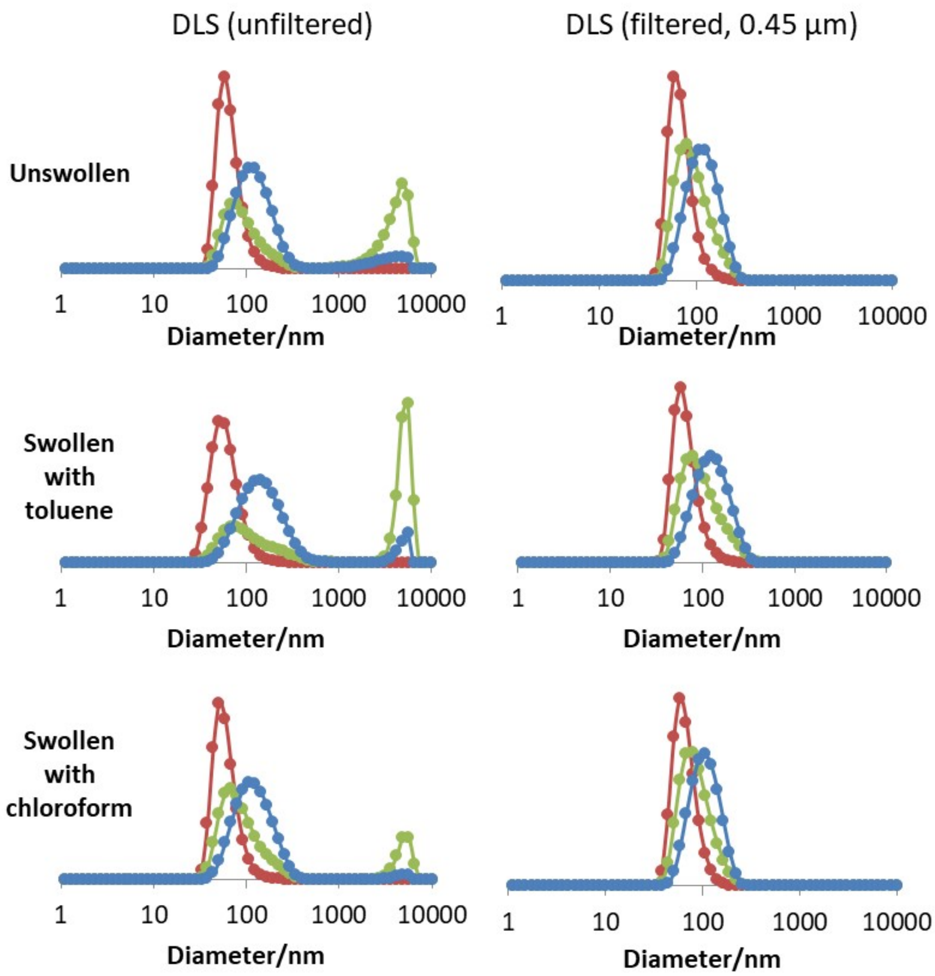

Figure S 6. Comparison of unfiltered and filtered DLS traces of the aqueous dispersions of the CCMs R_0-$(SS^{-}Na^{+})_{140}$-*b*-St_{50}-*b*-$(St_{0.95}$-*co*-$DPPS_{0.05})_{300}$-*b*-$(St_{0.90}$-*co*-$DEGDMA_{0.10})_{150}$-SC(S)S*n*Pr. Color coding for the DLS size distributions: number (red), volume (green) and intensity (blue).

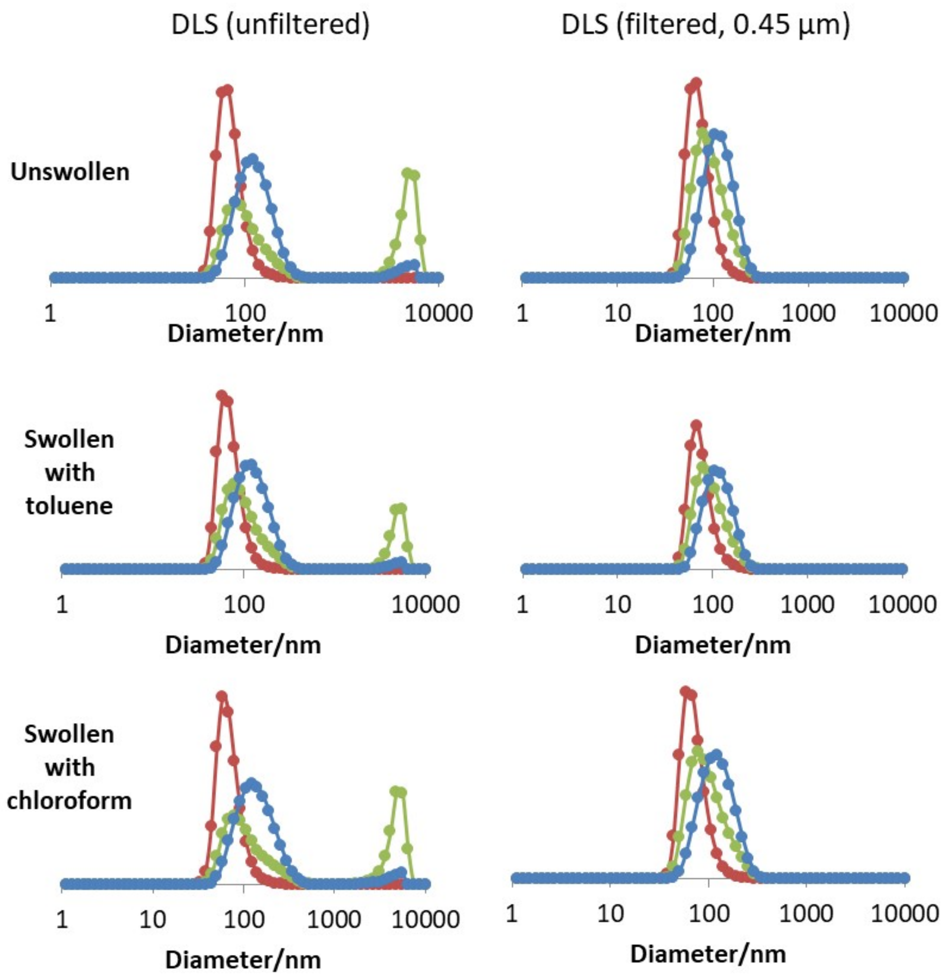

Figure S 7. Comparison of unfiltered and filtered DLS traces of the aqueous dispersions of the CCMs R_0-$(SS^{-}Na^{+})_{140}$-*b*-St_{50}-*b*-$(St_{0.90}$-*co*-$DPPS_{0.10})_{300}$-*b*-$(St_{0.90}$-*co*-$DEGDMA_{0.10})_{150}$-SC(S)S*n*Pr. Color coding for the DLS size distributions: number (red), volume (green) and intensity (blue).

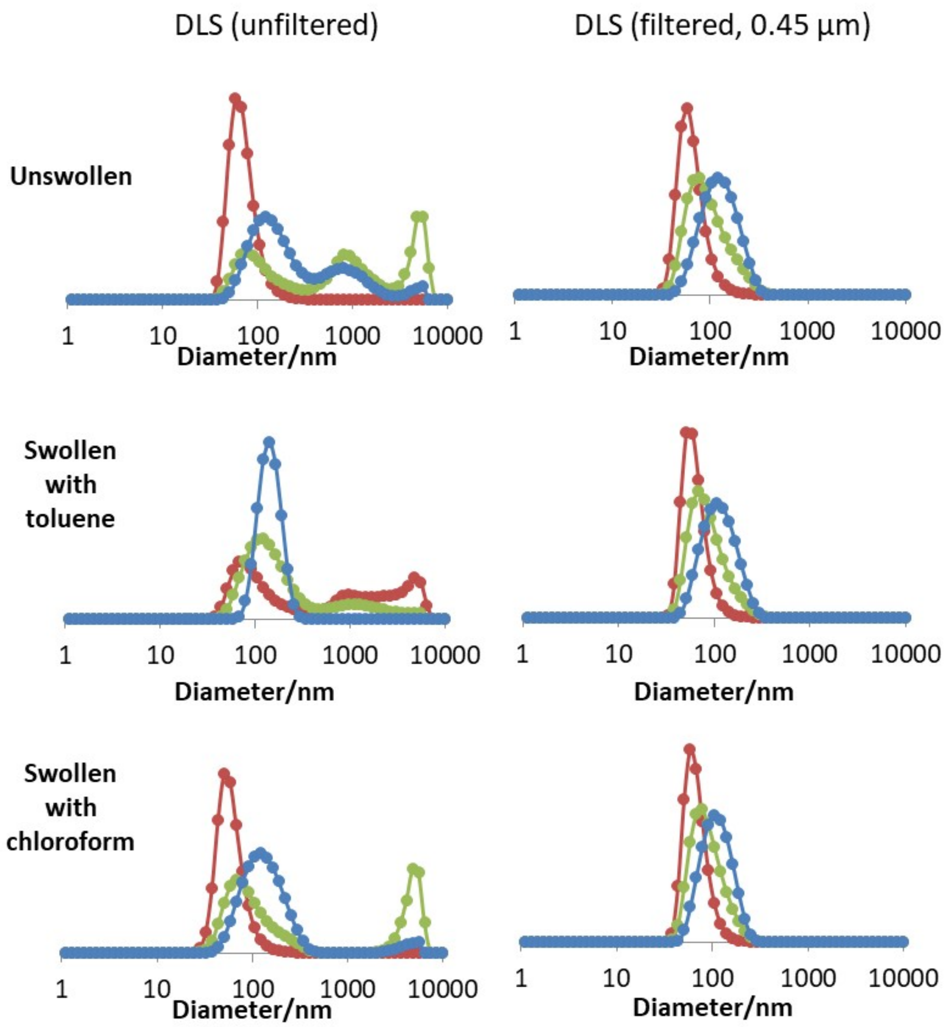

Figure S 8. Comparison of unfiltered and filtered DLS traces of the aqueous dispersions of the CCMs R_0-$(SS^-Na^+)_{140}$-*b*-St_{50}-*b*-$(St_{0.80}$-*co*-$DPPS_{0.20})_{300}$-*b*-$(St_{0.90}$-*co*-$DEGDMA_{0.10})_{150}$-SC(S)S*n*Pr. Color coding for the DLS size distributions: number (red), volume (green) and intensity (blue).

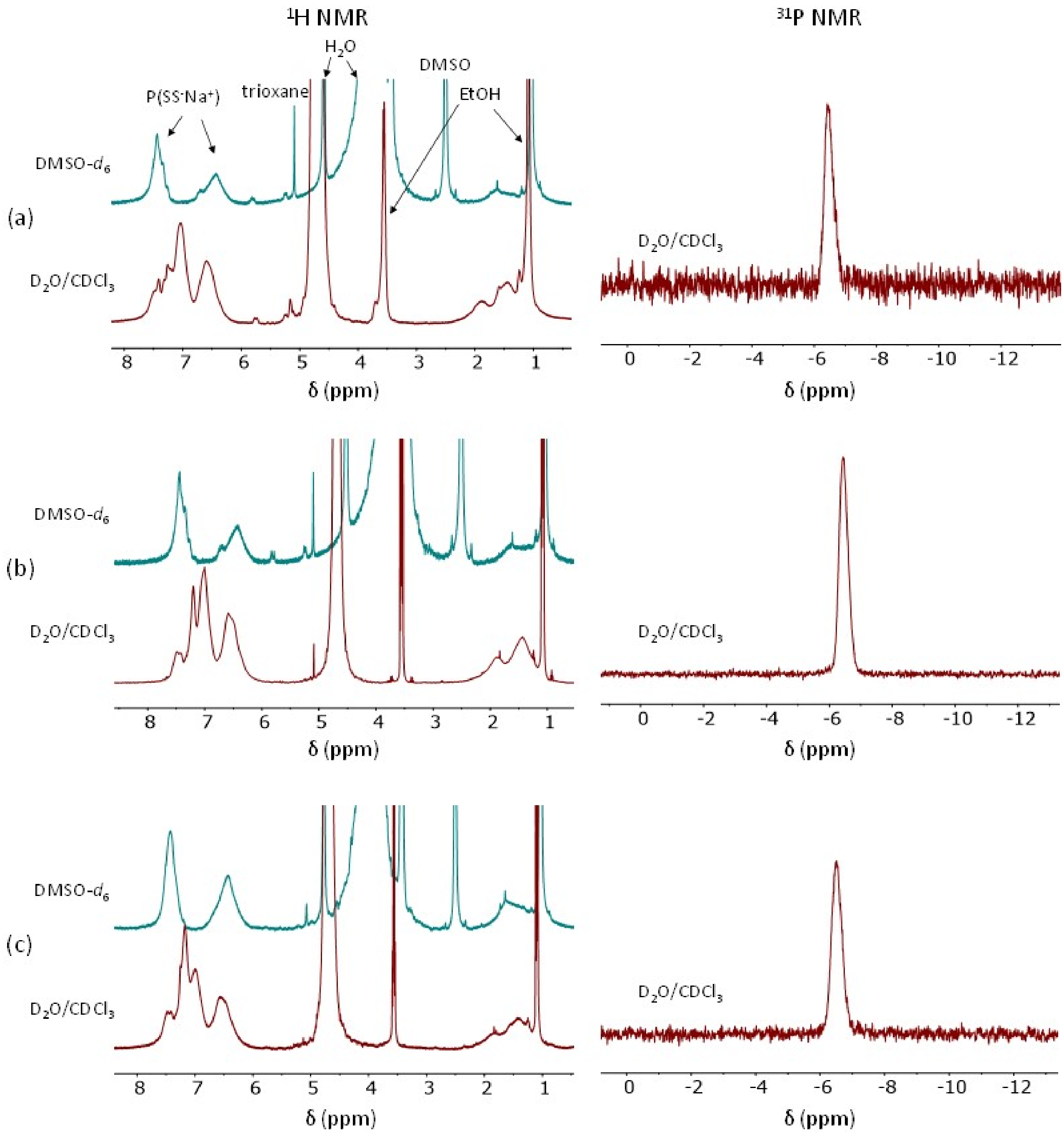

Figure S 9. ¹H and ³¹P NMR spectra of R0-(SS⁻Na⁺)140-*b*-St50-*b*-(St1-y-*co*-DPPSy)300-*b*-(St0.9-*co*-DEGDMA0.1)150-SC(S)S*n*Pr latexes in $D_2O/CDCl_3$ (3:1 v/v). (a) y = 0.05; (b) y = 0.10; (c) y = 0.20. The ¹H NMR spectra in DMSO-*d6* are also shown for comparison.

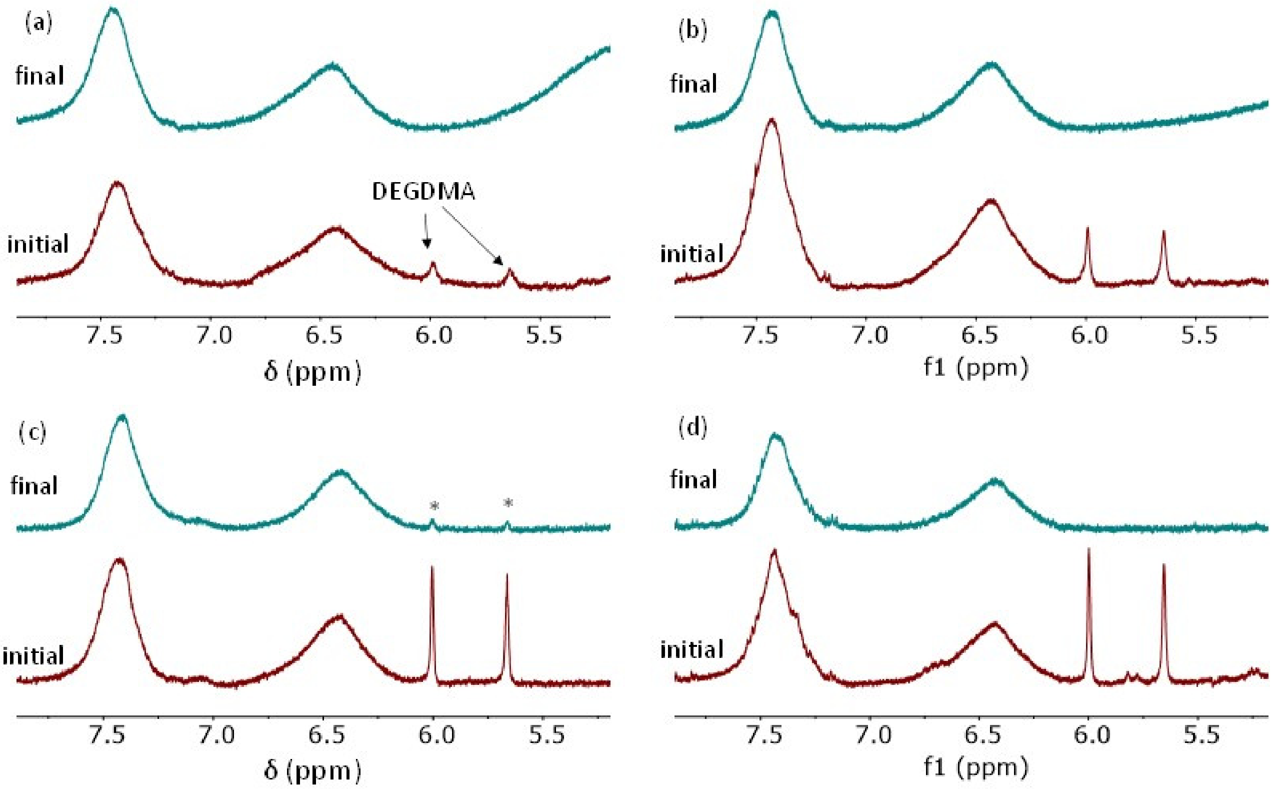

Figure S 10. ^{1}H NMR monitoring of the DEGDMA polymerization for the crosslinking of R_0-$(SS^-Na^+)_{140}$-*b*-$(St_{1-y}$-*co*-$DPPS_y)_{300}$-SC(S)S*n*Pr to yield the CCMs R_0-$(SS^-Na^+)_{140}$-*b*-$(St_{1-y}$-*co*-$DPPS_y)_{300}$-*b*-$DEGDMA_{15}$-SC(S)S*n*Pr. (a) y = 0.05; (b) y = 0.10; (c) y = 0.20; (d) y = 0.25. The starred resonances correspond to residual DEGDMA (< 1% by integration against the trioxane standard). All NMR samples were prepared by adding a drop of the reaction mixture directly to the DMSO-d_6 solvent in the NMR tube.

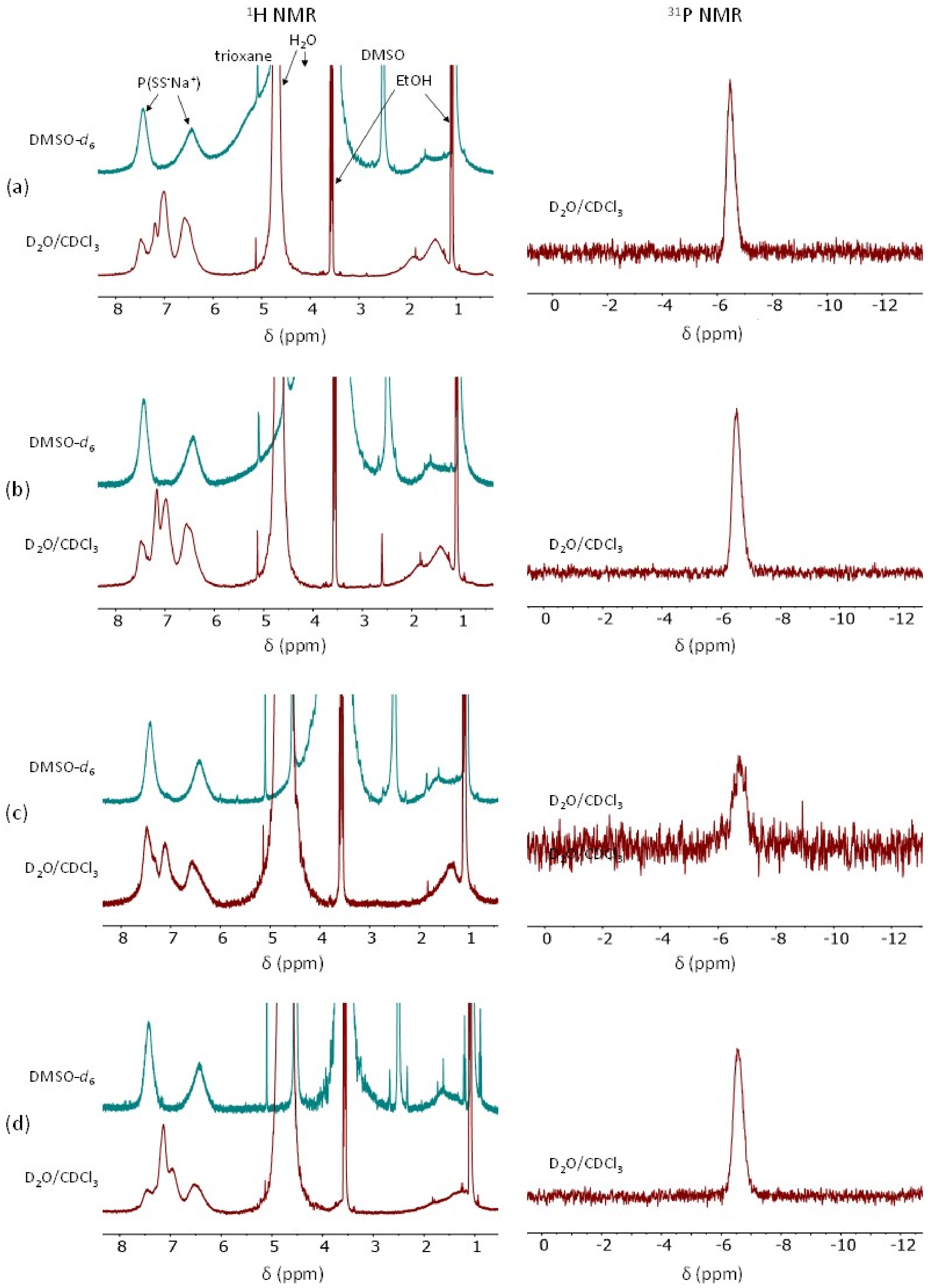

Figure S 11. ^{1}H and ^{31}P NMR spectra of R_0-$(SS^{-}Na^{+})_{140}$-*b*-$(St_{1-y}$-*co*-$DPPS_y)$-*b*-$DEGDMA_{15}$-SC(S)S*n*Pr latexes in $D_2O/CDCl_3$ (3:1 v/v). (a) y = 0.05; (b) y = 0.1; (c) y = 0.2; (d) y = 0.25. The ^{1}H NMR spectra in DMSO-d_6 are also shown for comparison.

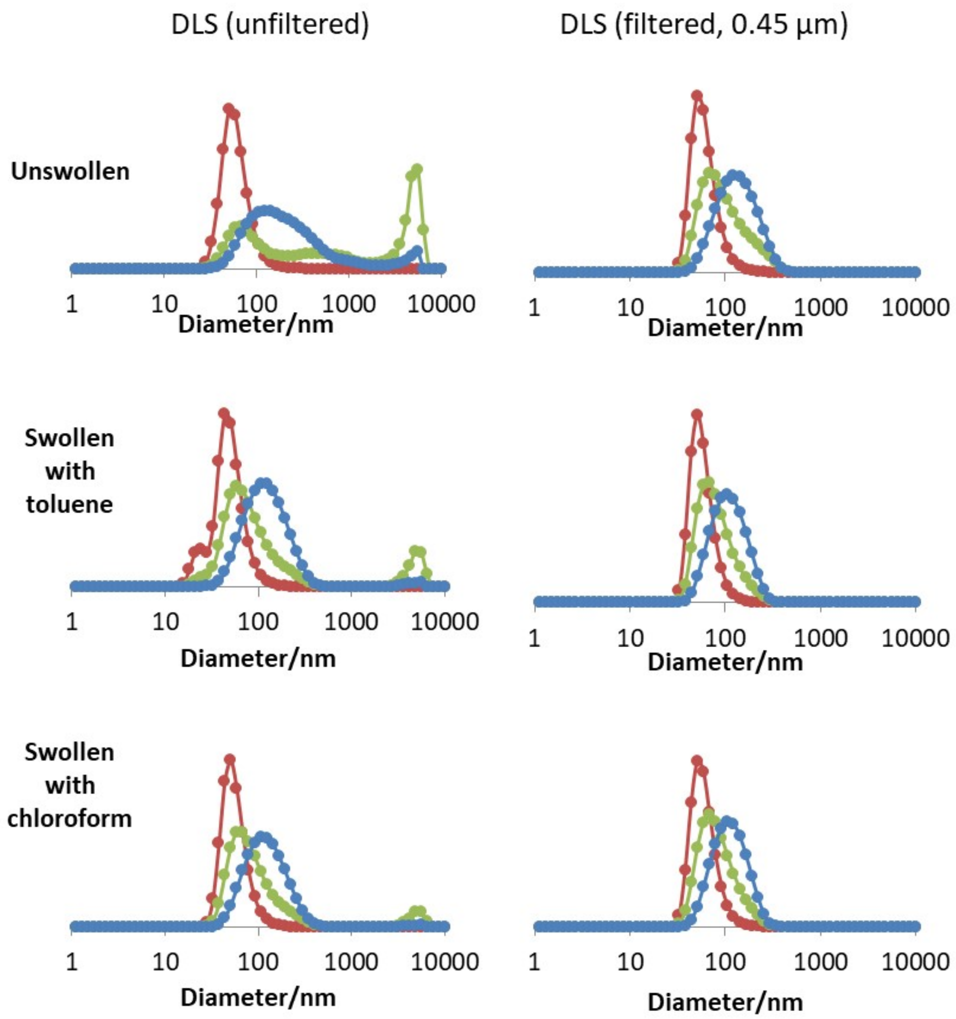

Figure S 12. Comparison of unfiltered and filtered DLS traces of the aqueous dispersions of the CCMs R$_0$-(SS$^-$Na$^+$)$_{140}$-*b*-(St$_{0.95}$-*co*-DPPS$_{0.05}$)$_{300}$-*b*-DEGDMA$_{15}$-SC(S)S*n*Pr. Color coding for the DLS size distributions: number (red), volume (green) and intensity (blue).

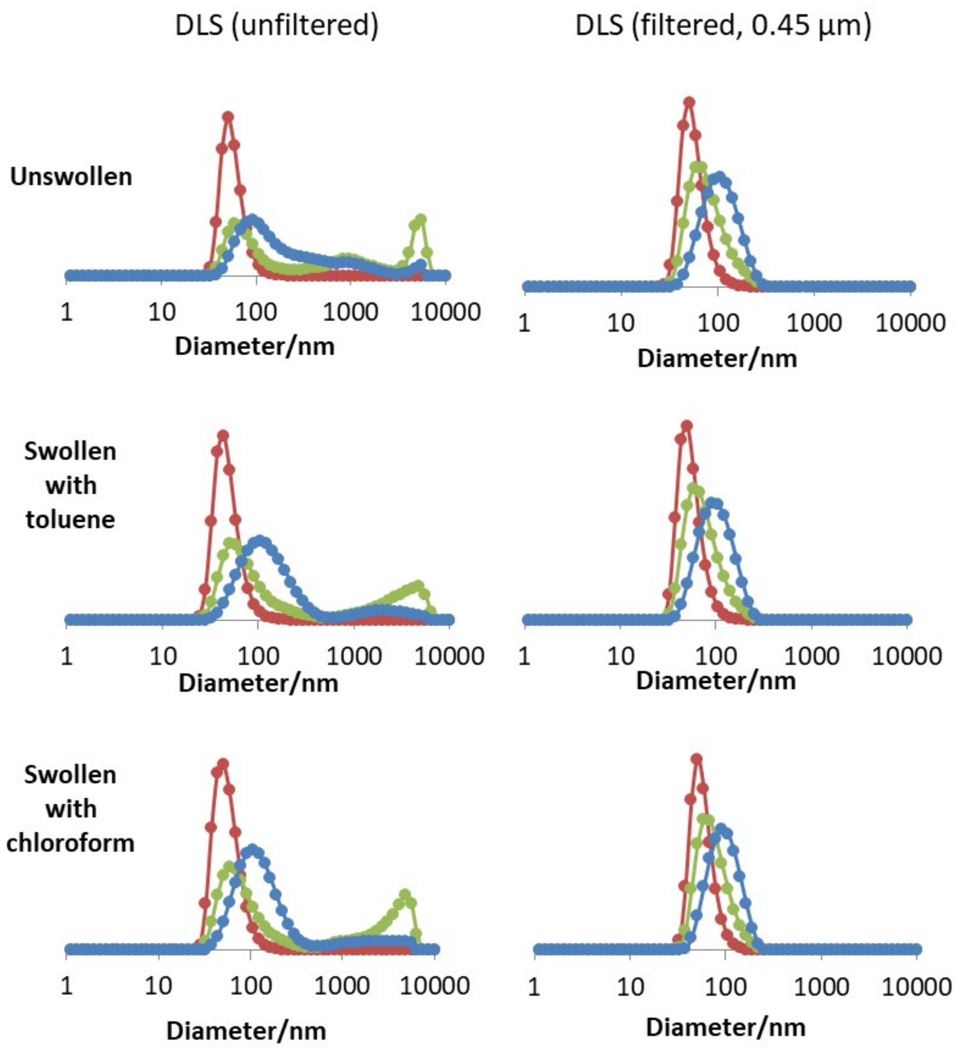

Figure S 13. Comparison of unfiltered and filtered DLS traces of the aqueous dispersions of the CCMs R_0-$(SS^-Na^+)_{140}$-*b*-$(St_{0.90}$-*co*-$DPPS_{0.10})_{300}$-*b*-$DEGDMA_{15}$-SC(S)S*n*Pr. Color coding for the DLS size distributions: number (red), volume (green) and intensity (blue).

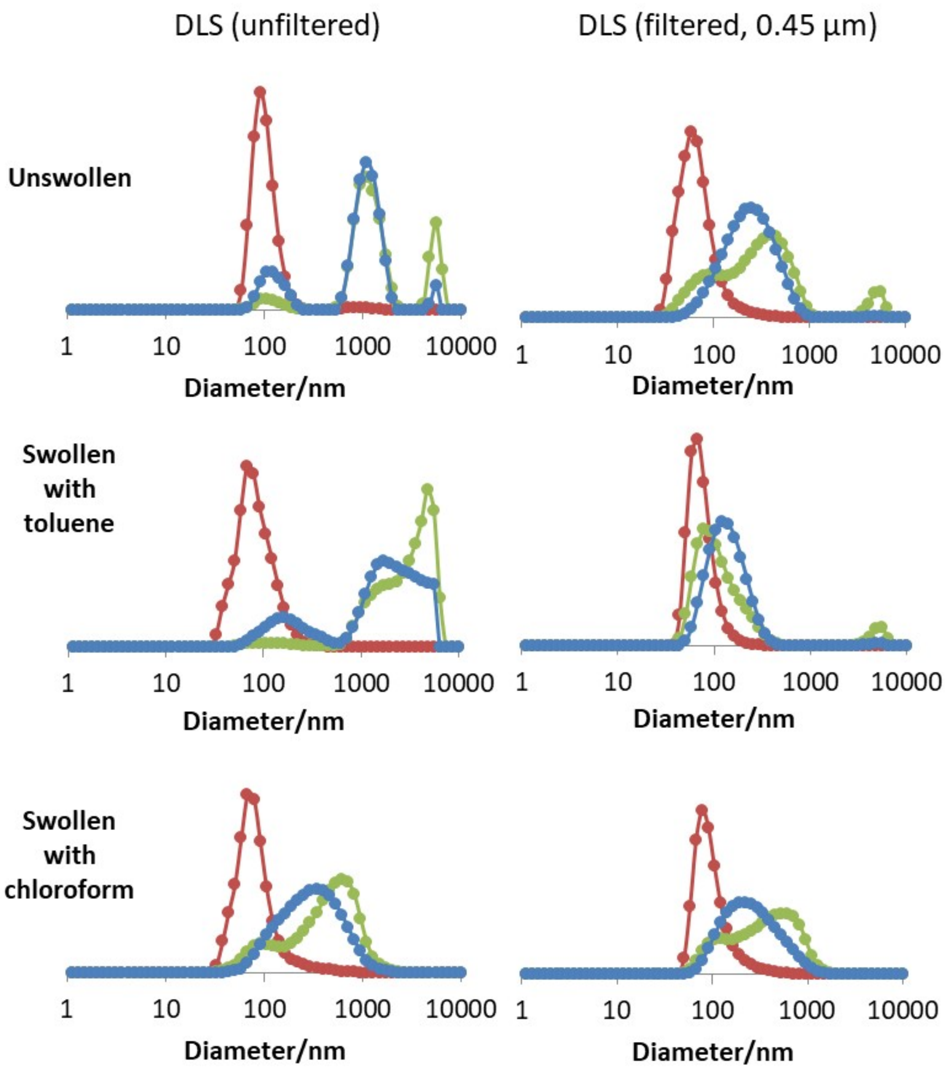

Figure S 14. Comparison of unfiltered and filtered DLS traces of the aqueous dispersions of the CCMs R_0-$(SS^{-}Na^{+})_{140}$-*b*-$(St_{0.80}$-*co*-$DPPS_{0.20})_{300}$-*b*-$DEGDMA_{15}$-SC(S)S*n*Pr. Color coding for the DLS size distributions: number (red), volume (green) and intensity (blue).

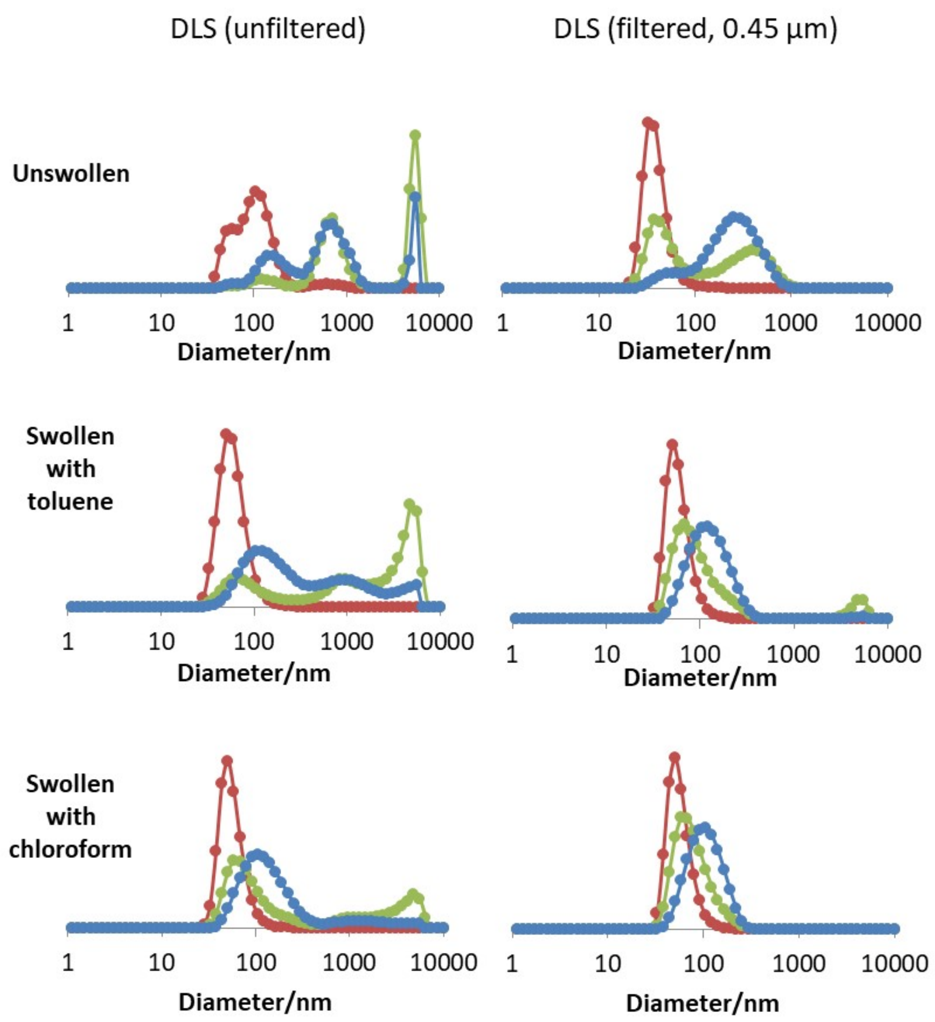

Figure S 15. Comparison of unfiltered and filtered DLS traces of the aqueous dispersions of the CCMs R_0-$(SS^-Na^+)_{140}$-*b*-$(St_{0.75}$-*co*-$DPPS_{0.25})_{300}$-*b*-$DEGDMA_{15}$-SC(S)S*n*Pr. Color coding for the DLS size distributions: number (red), volume (green) and intensity (blue).

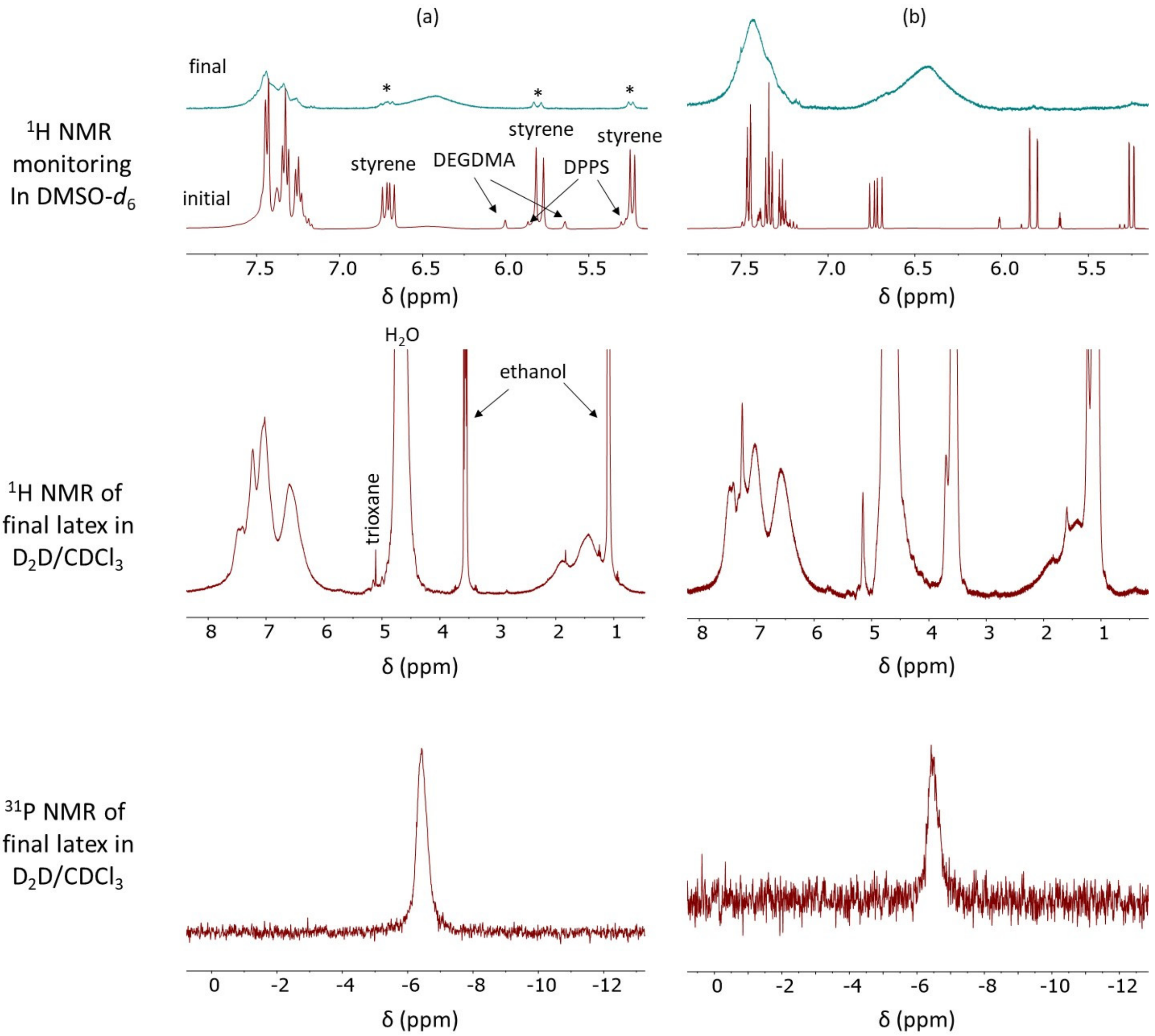

Figure S 16. NMR data for the synthesis of the NGs R_0-$(SS^-Na^+)_{140}$-*b*-St_x-*b*-(St_y-*co*-$DPPS_z$-*co*-$DEGDMA_{15}$)-SC(S)S*n*Pr. (a) x = 0, y = 285, z = 15; (b) x = 50, y = 425, z = 30. The starred resonances correspond to residual styrene [2.2% for (a), < 1% for (b), by integration against the trioxane standard]. All NMR samples were prepared by adding a drop of the reaction mixture directly to the deuterated solvent or solvent mixture in the NMR tube.

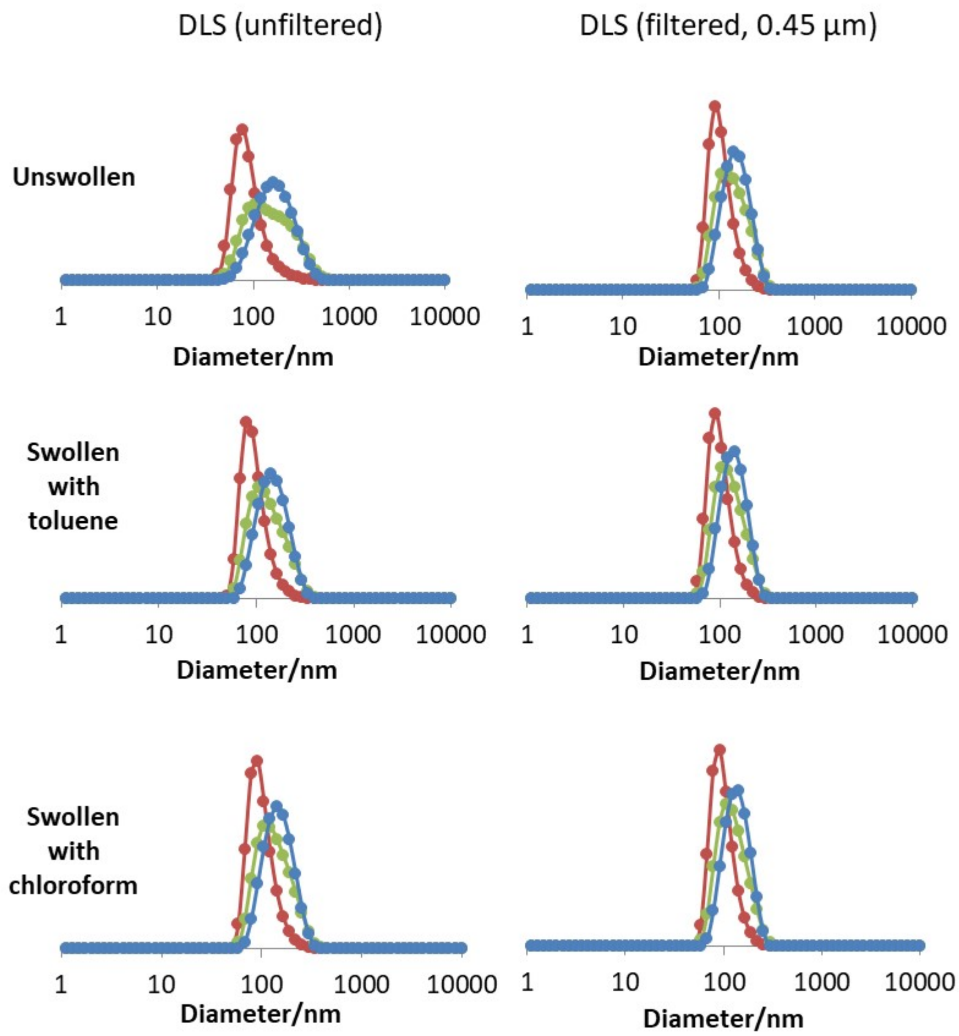

Figure S 17. Comparison of unfiltered and filtered DLS traces of the aqueous dispersions of the NG R_0-(SS^-Na^+)$_{140}$-*b*-(St_{285}-*co*-$DPPS_{15}$-*co*-$DEGDMA_{15}$)-SC(S)S*n*Pr. Color coding for the DLS size distributions: number (red), volume (green) and intensity (blue).

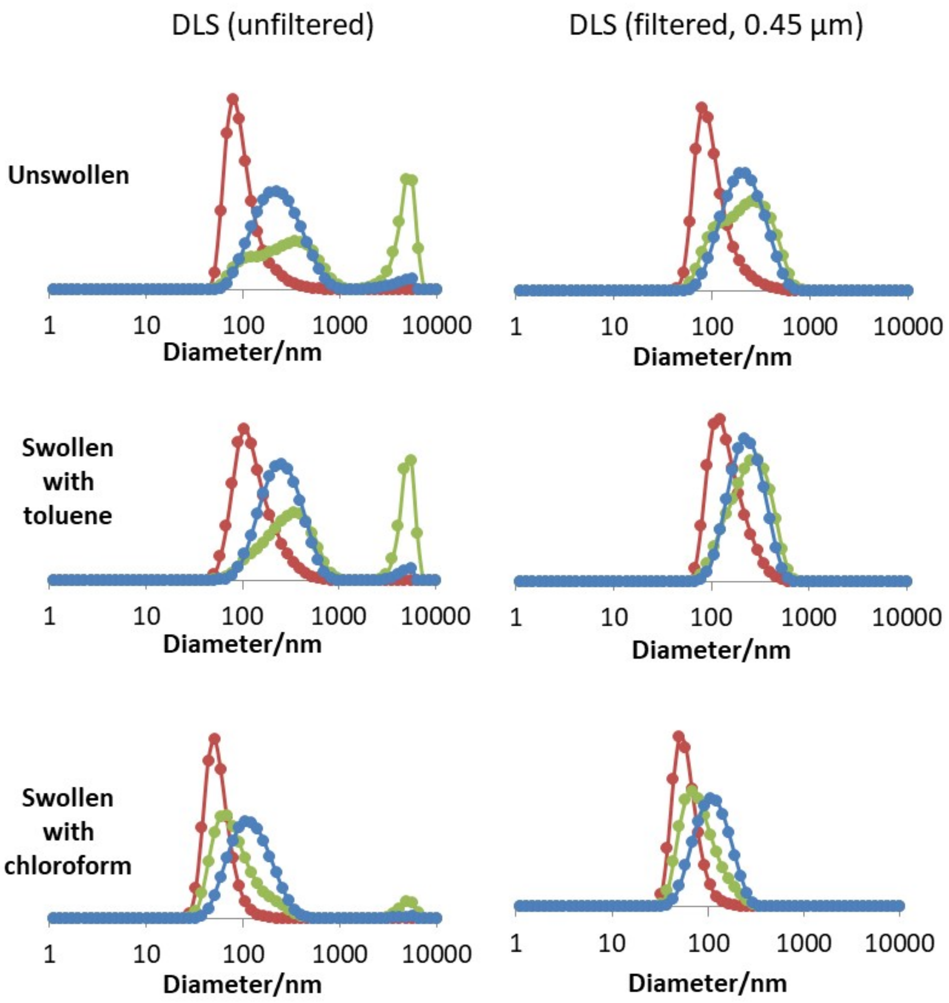

Figure S 18. Comparison of unfiltered and filtered DLS traces of the aqueous dispersions of the NG R_0-(SS^-Na^+)$_{140}$-*b*-St_{50}-*b*-(St_{425}-*co*-$DPPS_{30}$-*co*-$DEGDMA_{15}$)-SC(S)S*n*Pr. Color coding for the DLS size distributions: number (red), volume (green) and intensity (blue).

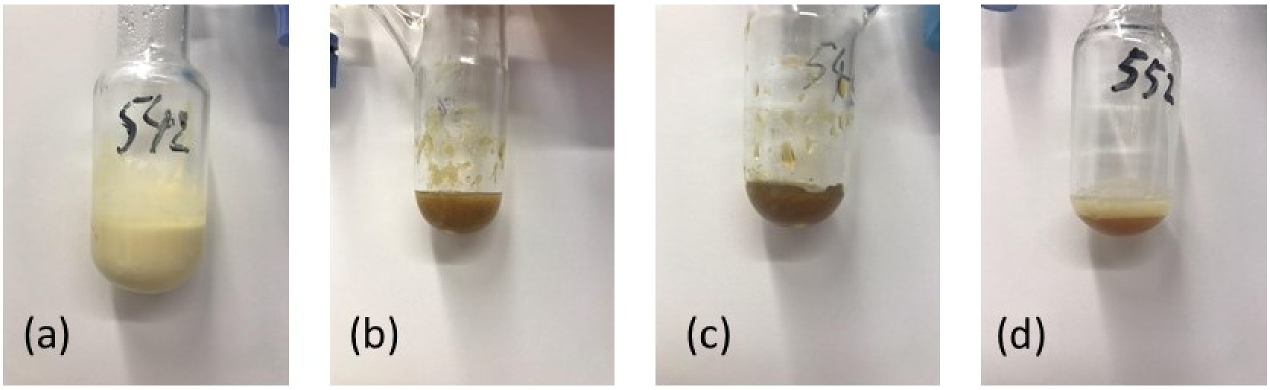

Figure S 19. Photos of the CCM and NG polymer latexes after treatment with a toluene solution of $[RhCl(COD)]_2$ (P/Rh = 1:1). (a) R_0-(SS^-Na^+)$_{140}$-*b*-($St_{0.75}$-*co*-$DPPS_{0.25}$)$_{300}$-*b*-$DEGDMA_{15}$-SC(S)S*n*Pr; (b) R_0-(SS^-Na^+)$_{140}$-*b*-($St_{0.9}$-*co*-$DPPS_{0.1}$)$_{300}$-*b*-$DEGDMA_{15}$-SC(S)S*n*Pr; (c) R_0-(SS^-Na^+)$_{140}$-*b*-St_{50}-*b*-($St_{0.9}$-*co*-$DPPS_{0.1}$)$_{300}$-*b*-($St_{0.9}$-*co*-$DEGDMA_{0.1}$)$_{150}$-SC(S)S*n*Pr; (d) R_0-(SS^-Na^+)$_{140}$-*b*-(St_{285}-*co*-$DPPS_{15}$-*co*-$DEGDMA_{15}$)-SC(S)S*n*Pr.

(b) ^{1}H NMR investigations of the Rh-sulfonate interaction

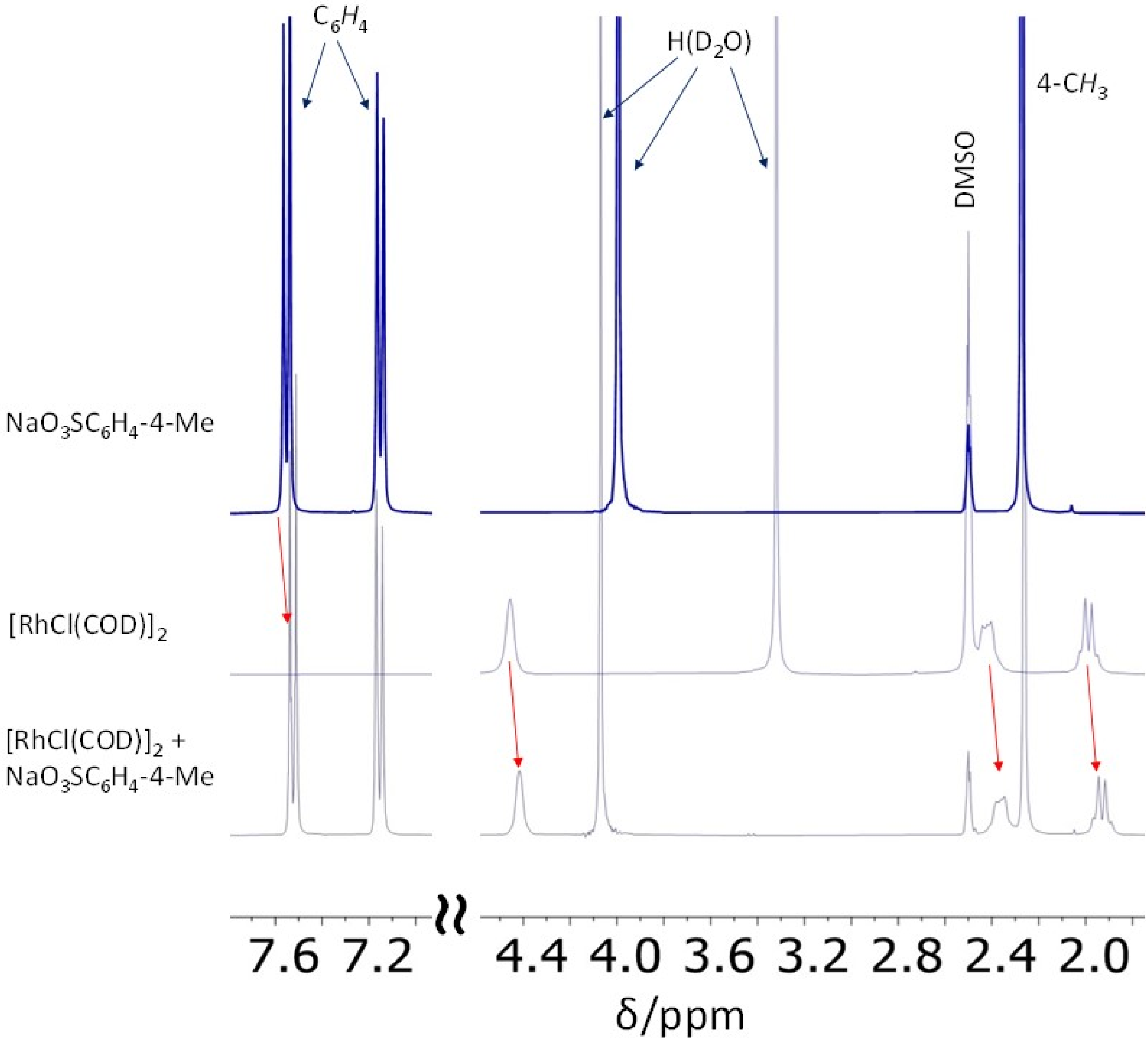

Figure S 20. ^{1}H NMR spectrum in DMSO-d_6/D_2O (4:1 v/v) of the $[RhCl(COD)]_2$/$NaO_3SC_6H_4$-4-CH_3 mixture (Rh/sulfonate = ca. 1:5), in comparison with those of the separate reagents measured under the same conditions.

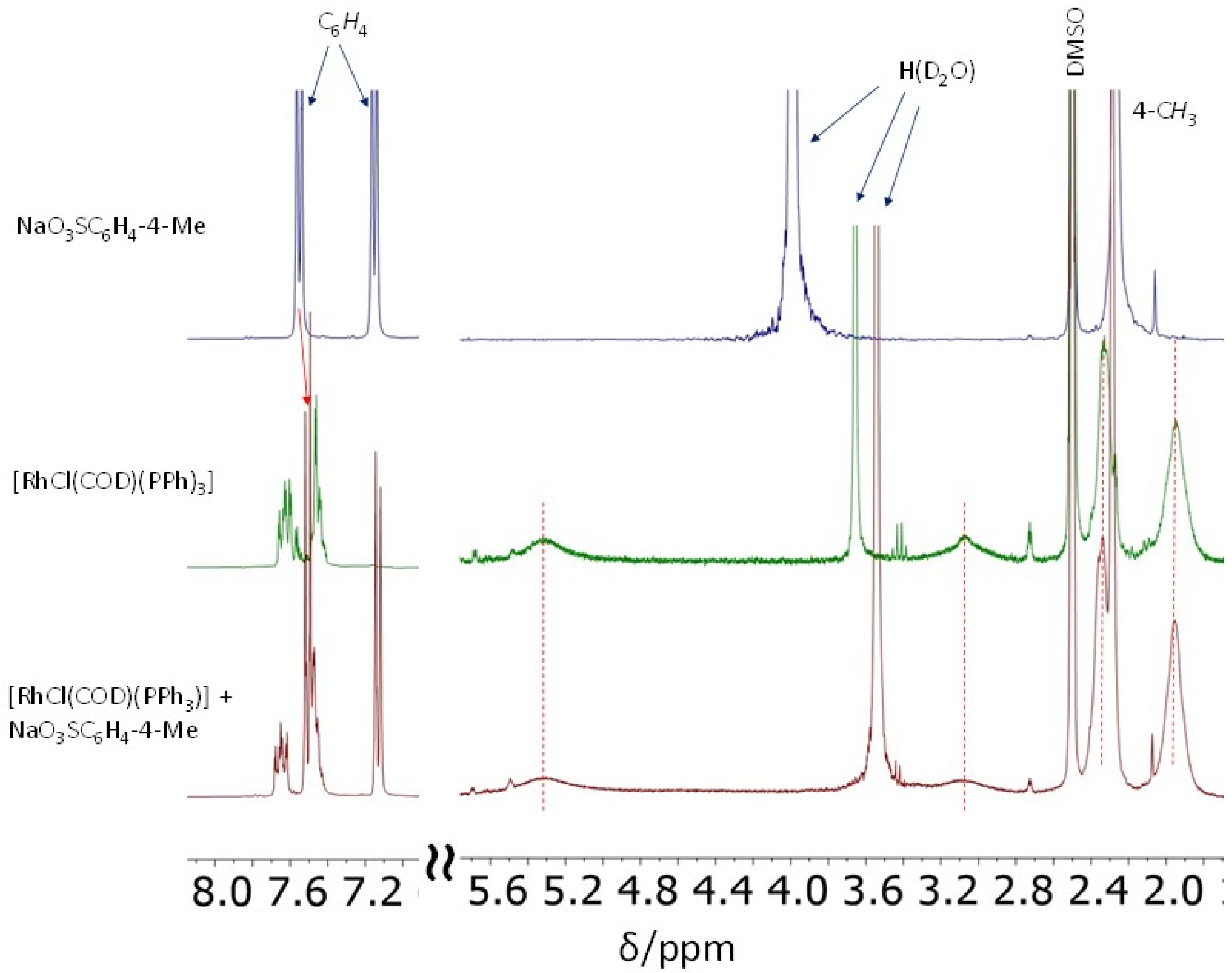

Figure S 21. ^{1}H NMR spectrum in DMSO-d_6/D_2O (4:1 v/v) of the [RhCl(COD)(PPh_3)]/$NaO_3SC_6H_4$-4-CH_3 mixture (Rh/sulfonate = ca. 1:5), in comparison with those of the separate reagents measured under the same conditions.

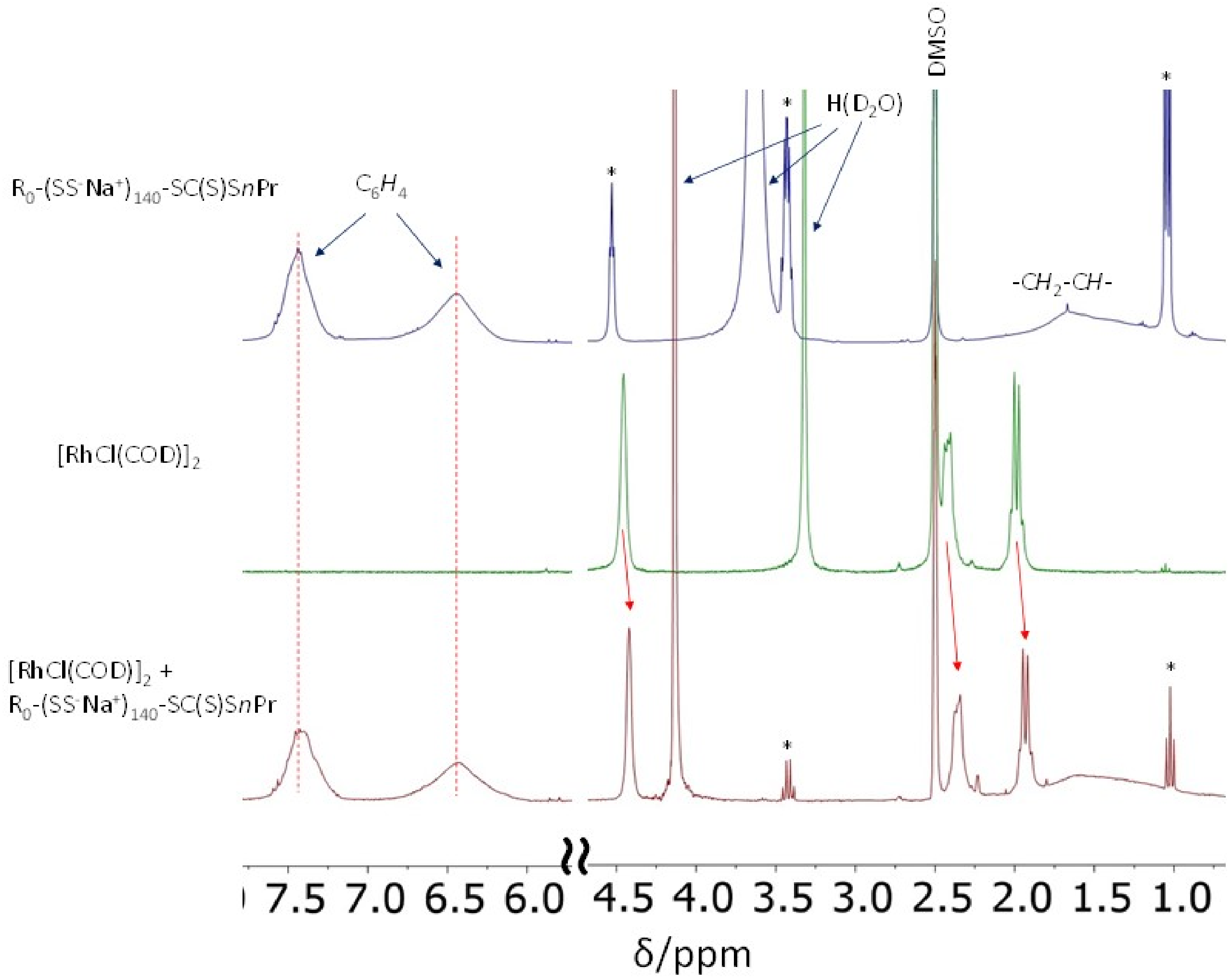

Figure S 22. ^{1}H NMR spectrum in DMSO-d_6/D_2O (6:1 v/v) of the $[RhCl(COD)]_2$/R_0-$(SS^{-}Na^{+})_{140}$-SC(S)S*n*Pr mixture (Rh/sulfonate = ca. 1:5), in comparison with those of the separate reagents measured under the same conditions. The starred resonances below to the residual ethanol in the macroRAFT polymer sample.

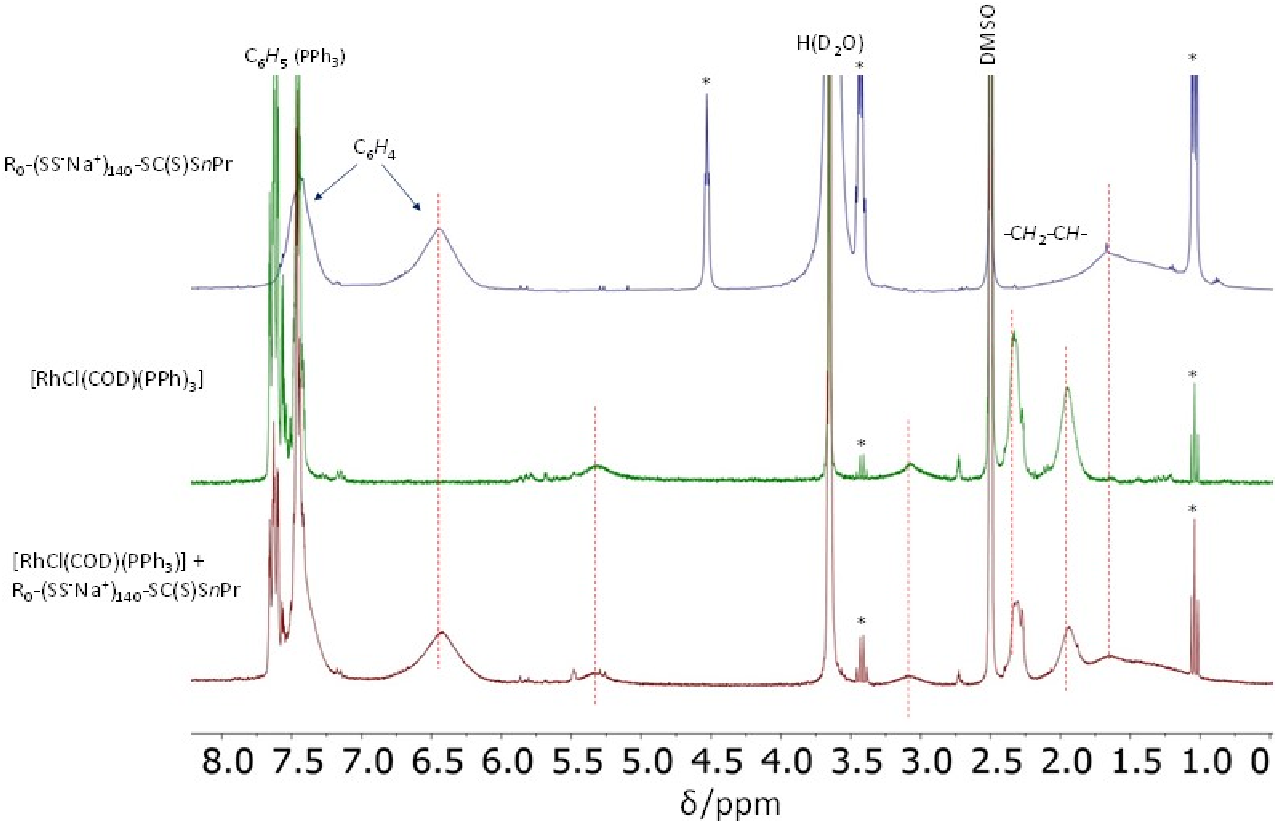

Figure S 23. ^{1}H NMR spectrum in DMSO-d_6/D_2O (6:1 v/v) of the [RhCl(COD)(PPh_3)]/R_0-(SS$^-$ Na$^+$)$_{140}$-SC(S)S*n*Pr mixture (Rh/sulfonate = ca. 1:5), in comparison with those of the separate reagents measured under the same conditions. The starred resonances below to the residual ethanol in the macroRAFT polymer sample.

(c) TPP-functionalized CCMs and NGs with P(SS$^-$Na$^+$-*co*-PEOMA) copolymer blocks in the hydrophilic shell

Table S 2. List of all polymers with a P(SS$^-$Na$^+$-*co*-PEOMA) shell synthesized in this study and reference to their characterization.

Formula [a]	SEC	^{1}H NMR	^{31}P NMR	DLS	TEM	ζ (mV)	Ref.
**1. Hydrosoluble R_0-(SS$^-$Na$^+$$_{0.2}$-*co*-PEOMA$_{0.8}$)$_x$-SC(S)SPr macroRAFT agents**							
R_0-[(SS$^-$Na$^+$)$_{0.2}$-*co*-PEOMA$_{0.8}$]$_{50}$-SC(S)S*n*Pr	Figure S 25	Figure S 24<br>Figure S 27		Figure S 26	Figure S 26	/	HW617
R_0-[(SS$^-$Na$^+$)$_{0.2}$-*co*-PEOMA$_{0.8}$]$_{140}$-SC(S)S*n*Pr	/	Figure S 24<br>Figure S 27		Figure S 26	Figure S 26	/	HW627
**2. Diblock P(SS$^-$Na$^+$-*co*-PEOMA)-*b*-St$_{50}$ macroRAFT agents**							
R_0-[(SS$^-$Na$^+$)$_{0.2}$-*co*-PEOMA$_{0.8}$]$_{50}$-*b*-St$_{50}$-SC(S)S*n*Pr	/	/		Figure S 28	Figure S 28	/	HW688
R_0-[(SS$^-$Na$^+$)$_{0.2}$-*co*-PEOMA$_{0.8}$]$_{140}$-*b*-St$_{50}$-SC(S)S*n*Pr	/	/		Figure S 28	Figure S 28	/	HW691
**3. Diblock P(SS$^-$Na$^+$-*co*-PEOMA)-*b*-P(St-*co*-DPPS) micelles**							
R_0-[(SS$^-$Na$^+$)$_{0.2}$-*co*-PEOMA$_{0.8}$]$_{50}$-*b*-(St$_{0.9}$-*co*-DPPS$_{0.1}$)$_{300}$-SC(S)S*n*Pr	/	Figure S 29	Figure S 29	Figure S 30<br>Figure S 35	Figure S 30	/	HW618HW701
R_0-[(SS$^-$Na$^+$)$_{0.2}$-*co*-PEOMA$_{0.8}$]$_{140}$-*b*-(St$_{0.9}$-*co*-DPPS$_{0.1}$)$_{300}$-SC(S)S*n*Pr		Figure S 29	Figure S 29	/	/	/	HW629
**4. CCMs with a P(SS$^-$Na$^+$-*co*-PEOMA) shell and a mixed DEGDMA-*co*-St core**							
R_0-[(SS$^-$Na$^+$)$_{0.2}$-*co*-PEOMA$_{0.8}$]$_{50}$-*b*-(St$_{0.9}$-*co*-DPPS$_{0.1}$)$_{300}$-*b*-(St$_{0.9}$-*co*-DEGDMA$_{0.1}$)$_{150}$-SC(S)S*n*Pr	/	Figure S 31	Figure S 31	Figure 10<br>Figure S 35	Figure 10	/	HW625
R_0-[(SS$^-$Na$^+$)$_{0.2}$-*co*-PEOMA$_{0.8}$]$_{140}$-*b*-(St$_{0.9}$-*co*-DPPS$_{0.1}$)$_{300}$-*b*-(St$_{0.9}$-*co*-DEGDMA$_{0.1}$)$_{150}$-SC(S)S*n*Pr	/	Figure S 31	Figure S 31	Figure 10	Figure 10	/	HW632
**5. CCM with P(PEOMA-*co*-SS$^-$Na$^+$) shell and neat DEGDMA core**							
R_0-(PSS$^-$Na$^+$$_{0.2}$-*co*-PEOMA$_{0.8}$)$_{50}$-*b*-(St$_{0.9}$-*co*-DPPS$_{0.1}$)$_{300}$-*b*-DEGDMA$_{90}$-SC(S)SPr	/	Figure S 32	Figure S 32<br>Figure 11	Figure 10	Figure 10	-25.9±4,5	HW620HW657
**6. NG with P(PEOMA-*co*-SS$^-$Na$^+$) shell**							
R_0-[(SS$^-$Na$^+$)$_{0.2}$-*co*-PEOMA$_{0.8}$]$_{50}$-*b*-St$_{50}$-*b*-(St$_{425}$-*co*-DPPS$_{30}$-*co*-DEGDMA$_{15}$)-SC(S)S*n*Pr	/	Figure S 33	Figure S 33	Figure S 34	Figure S 34	/	HW698
R_0-[(SS$^-$Na$^+$)$_{0.2}$-*co*-PEOMA$_{0.8}$]$_{140}$-*b*-St$_{50}$-*b*-(St$_{425}$-*co*-DPPS$_{30}$-*co*-DEGDMA$_{15}$)-SC(S)S*n*Pr	/	Figure S 33	Figure S 33	Figure S 34	Figure S 34	/	HW692

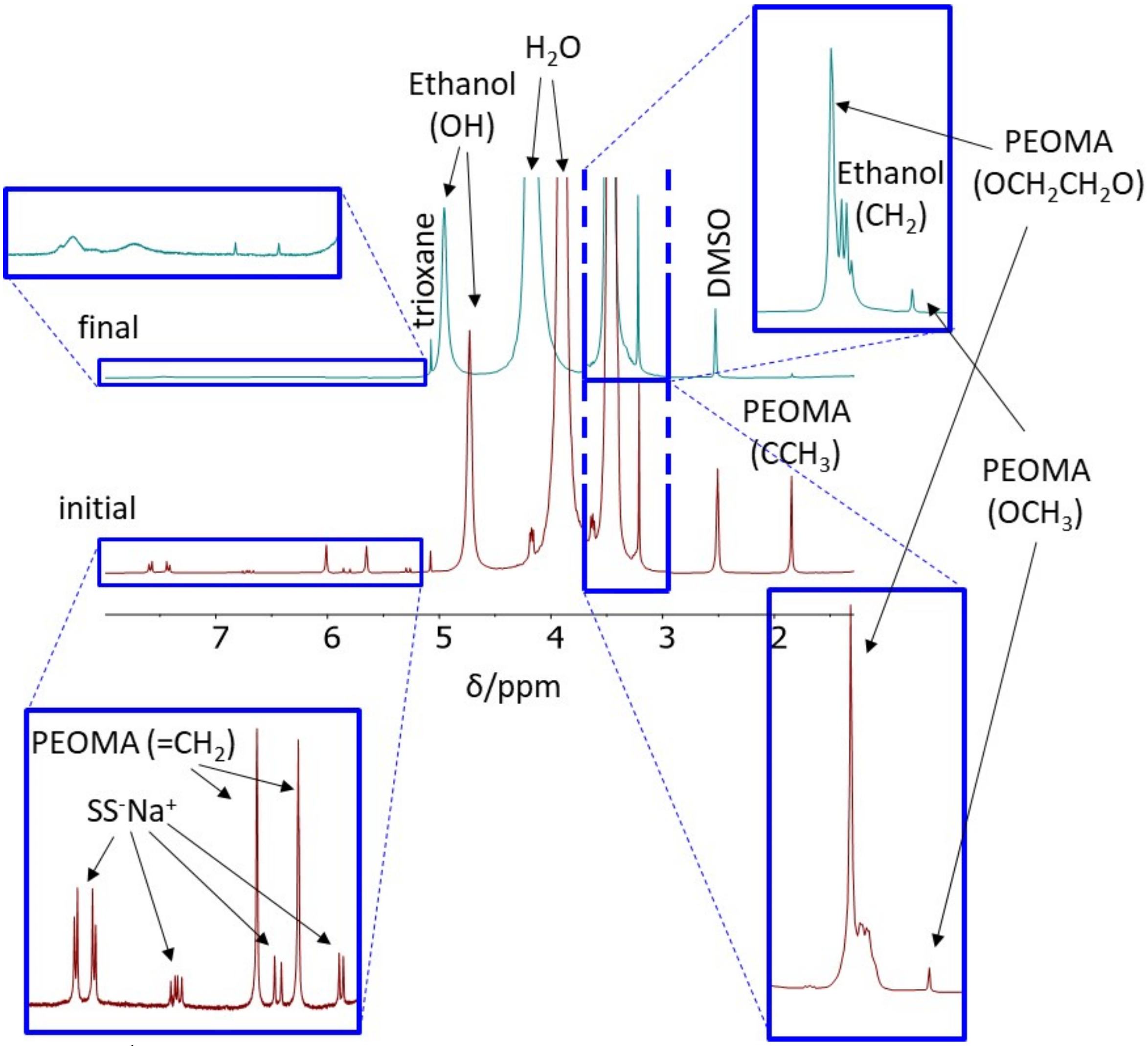

Figure S 24. ^{1}H NMR monitoring of the SS^-Na^+/PEOMA copolymerization for the synthesis of the R_0-[$(SS^-Na^+)_{0.2}$-*co*-$PEOMA_{0.8}$]$_{140}$-SC(S)S*n*Pr macroRAFT agents. All NMR samples were prepared by adding a drop of the reaction mixture directly to the DMSO-d_6 solvent in the NMR tube.

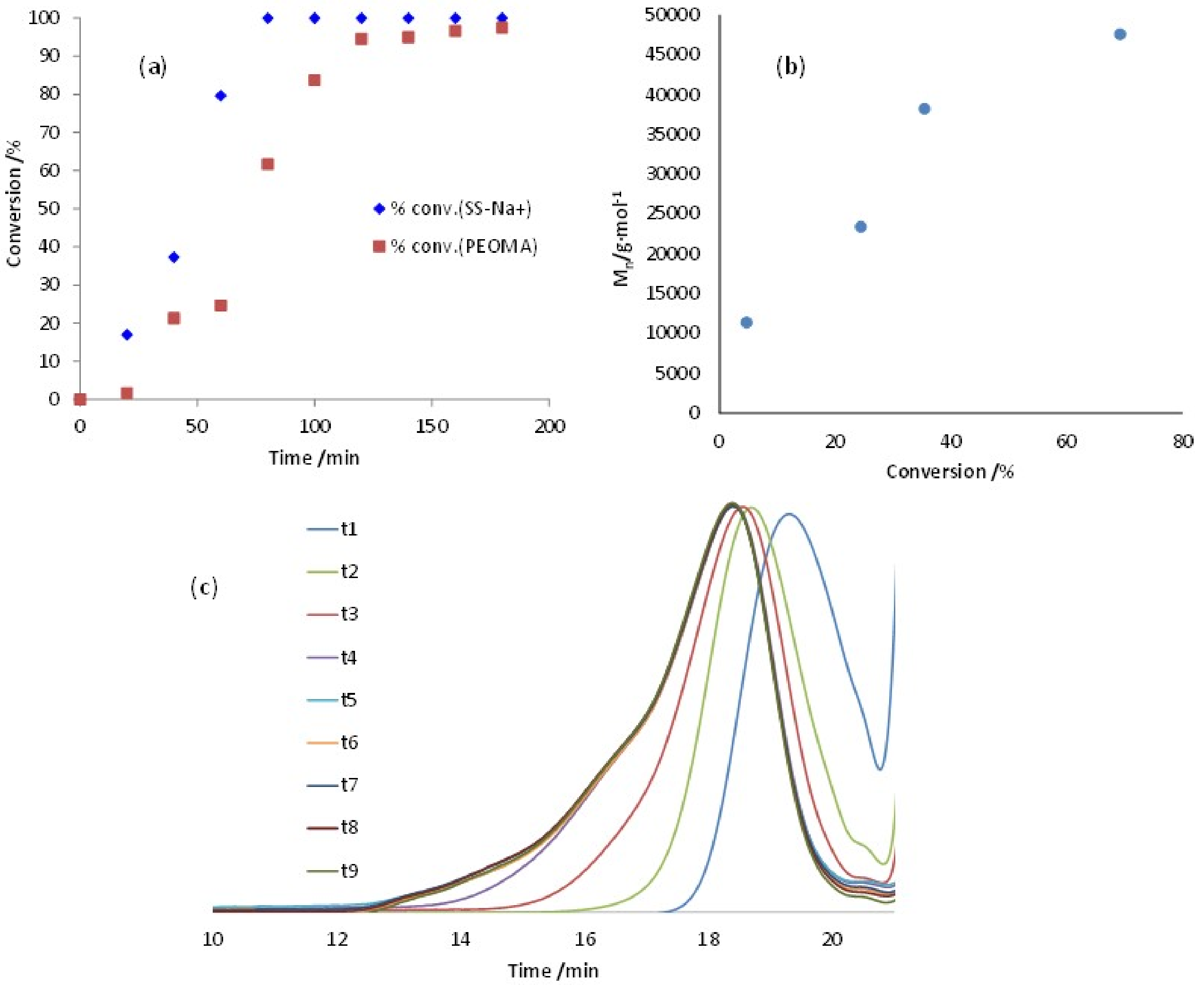

Figure S 25. ^{1}H NMR and SEC monitoring of the SS^-Na^+/PEOMA RAFT copolymerization: (a) individual monomer conversion *vs.* time; (b) evolution of the polymer molar mass with the global monomer conversion (c) GPC traces.

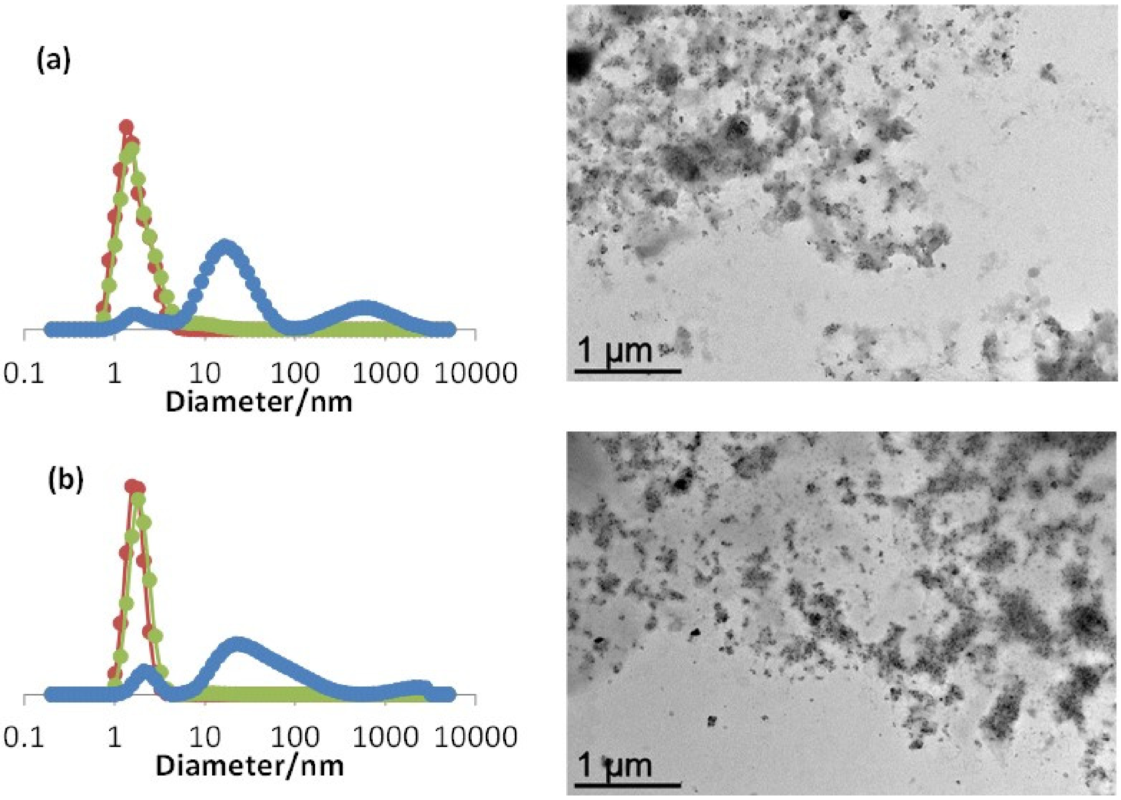

Figure S 26. DLS (left, unfiltered) and TEM (right) characterization of R_0-[$(SS^-Na^+)_{0.2}$-*co*-$PEOMA_{0.8}$]$_x$-SC(S)S*n*Pr. (a) x = 50; (b) x = 140. Color coding for the DLS size distributions: number (red), volume (green) and intensity (blue).

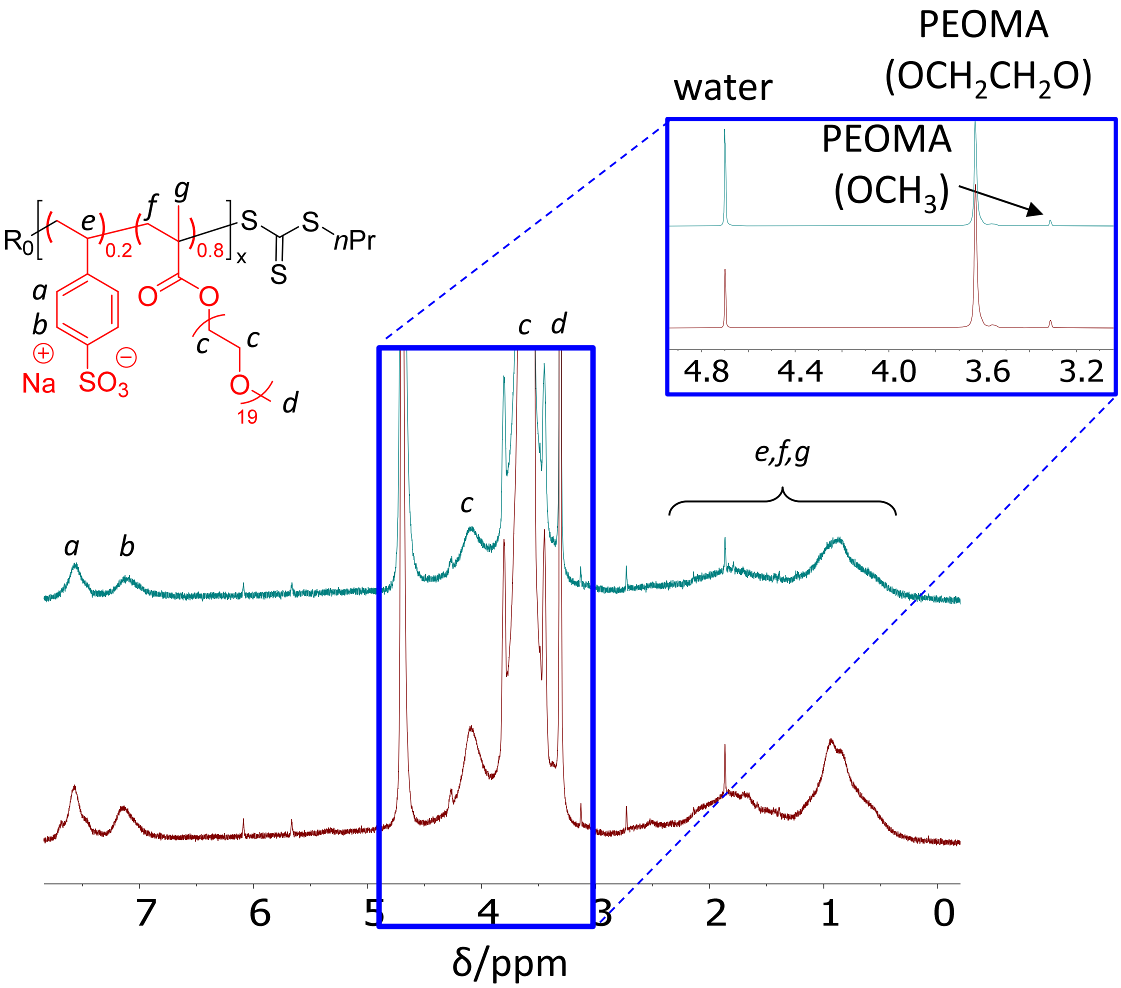

Figure S 27. ^{1}H NMR spectra of R0-[(SS⁻Na⁺)0.2-*co*-PEOMA0.8]x-SC(S)S*n*Pr. (a) x = 50 (red); (b) x = 140 (cyan) in D_2O.

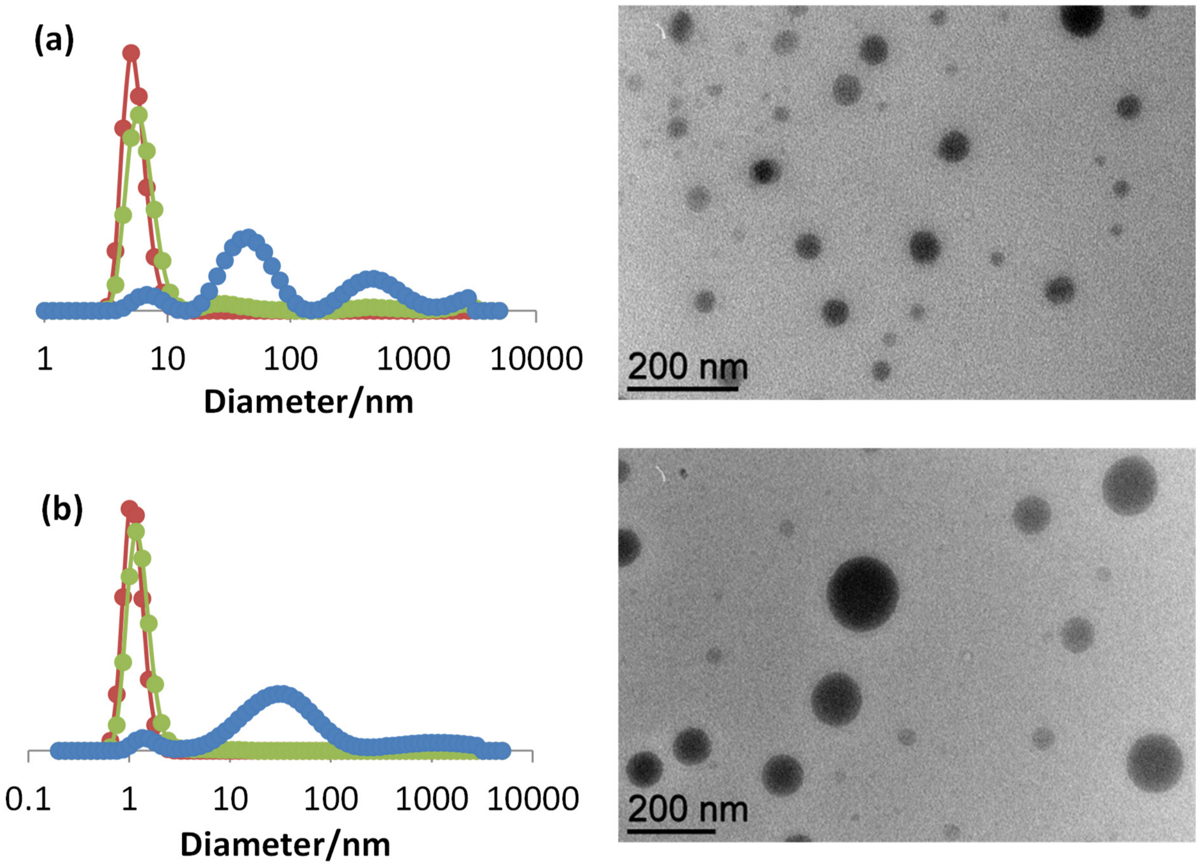

Figure S 28. DLS (left, unfiltered) and TEM (right) characterization of R_0-[$(SS^-Na^+)_{0.2}$-*co*-$PEOMA_{0.8}$]$_x$-*b*-St_{50}-SC(S)S*n*Pr. (a) x = 50; (b) x = 140. Color coding for the DLS size distributions: number (red), volume (green) and intensity (blue).

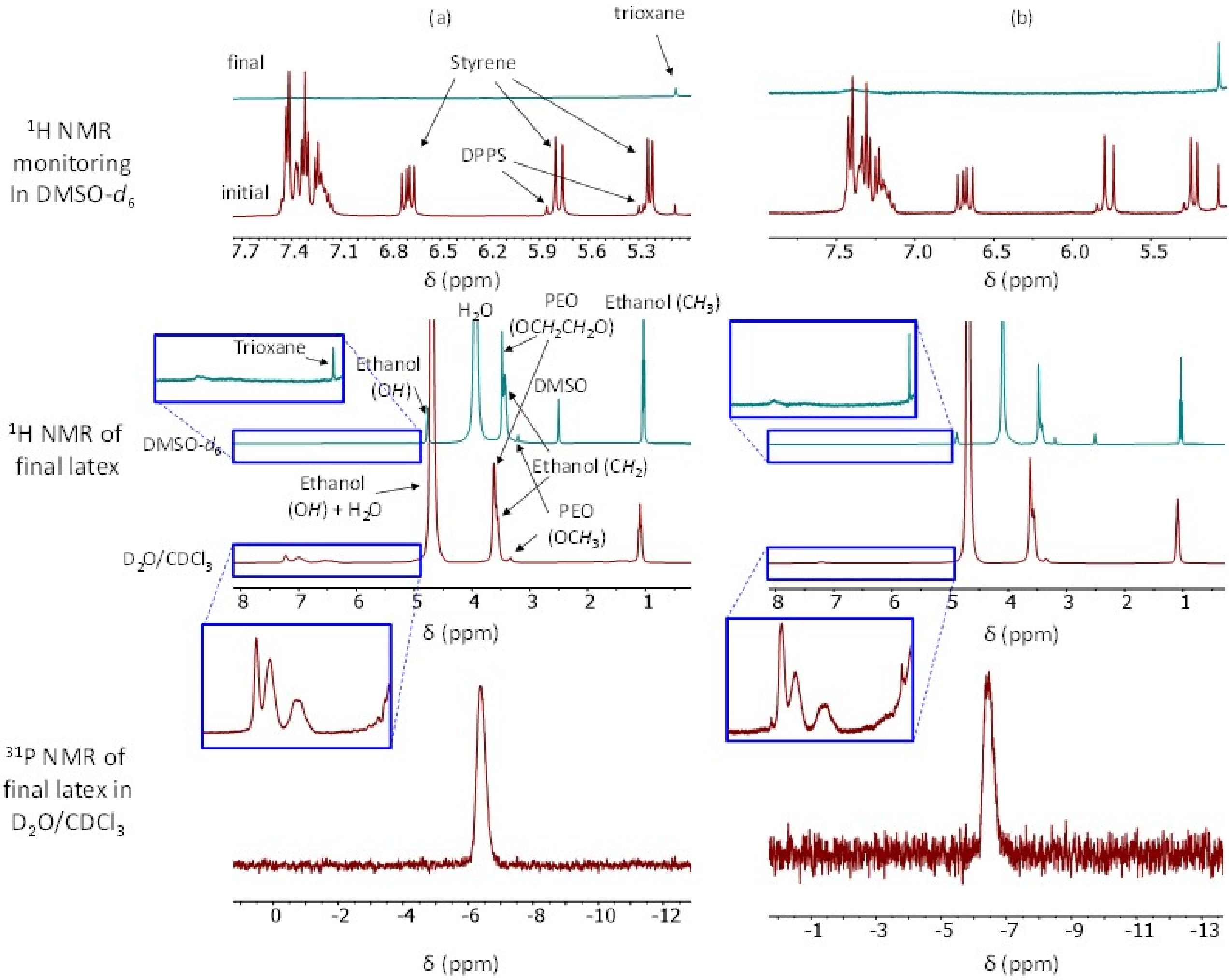

Figure S 29. NMR data for the synthesis of the R$_0$-[(SS$^-$Na$^+$)$_{0.2}$-*co*-PEOMA$_{0.8}$]$_x$-*b*-(St$_{0.9}$-*co*-DPPS$_{0.1}$)$_{300}$-SC(S)S*n*Pr diblock copolymer. (a) x = 50; (b) x = 140. All NMR samples were prepared by adding a drop of the reaction mixture directly to the deuterated solvent or solvent mixture in the NMR tube.

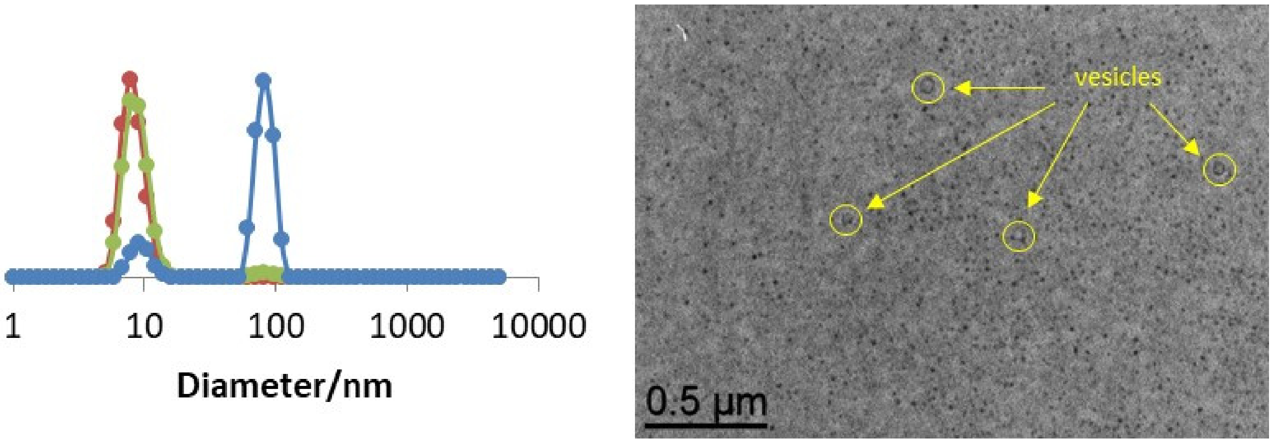

Figure S 30. DLS (left, unfiltered) and TEM (right) characterization of R$_0$-[(SS$^-$Na$^+$)$_{0.2}$-*co*-PEOMA$_{0.8}$]$_{50}$-*b*-(St$_{0.9}$-*co*-DPPS$_{0.1}$)$_{300}$-SC(S)S*n*Pr. Color coding for the DLS size distributions: number (red), volume (green) and intensity (blue).

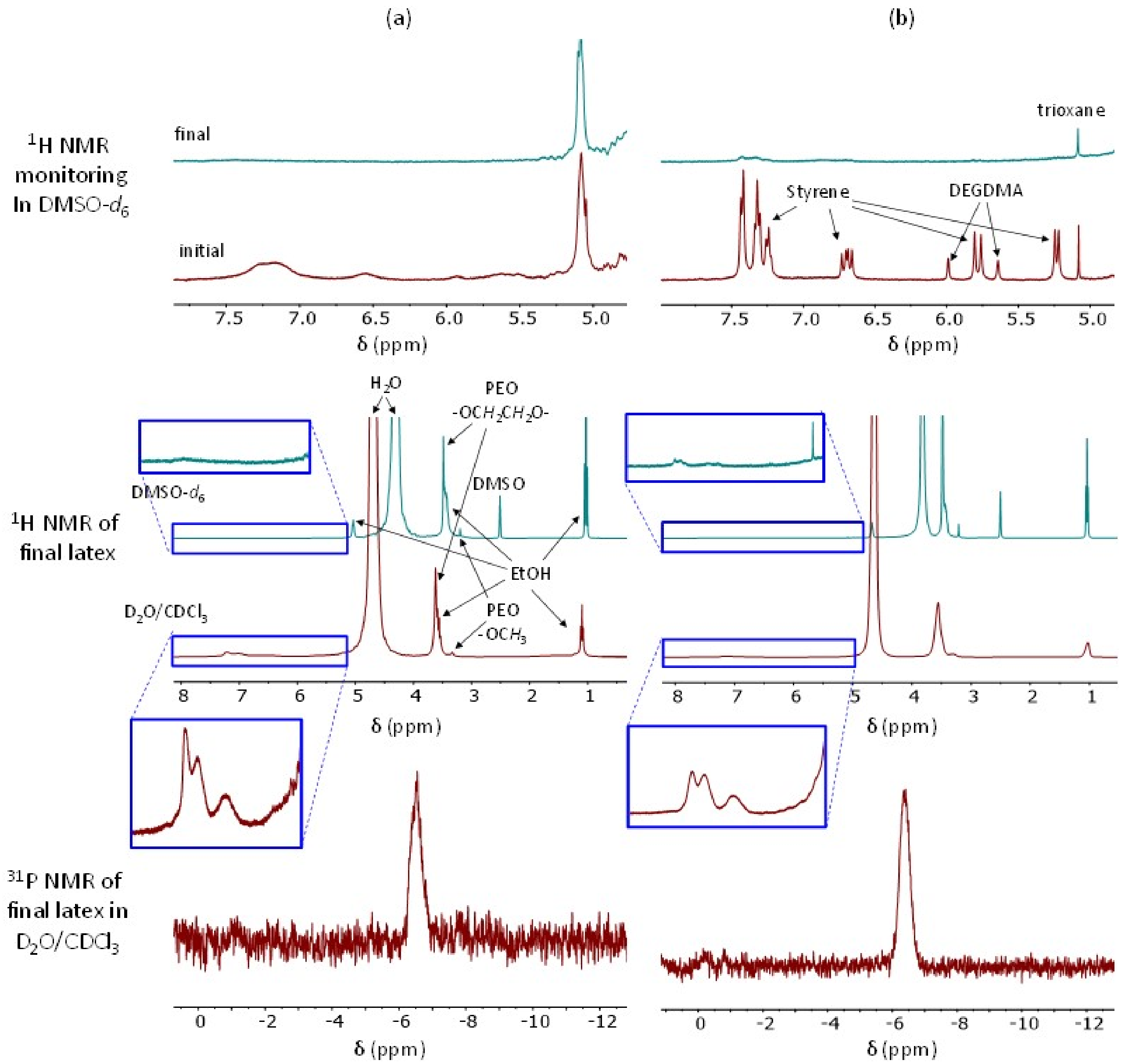

Figure S 31. NMR data for the synthesis of the R0-[(SS$^-$Na$^+$)$_{0.2}$-*co*-PEOMA$_{0.8}$]$_x$-*b*-(St$_{0.9}$-*co*-DPPS$_{0.1}$)$_{300}$-*b*-(St$_{0.9}$-*co*-DEGDMA$_{0.1}$)$_{150}$-SC(S)S*n*Pr CCMs. (a) x = 50; (b) x = 140. All NMR samples were prepared by adding a drop of the reaction mixture directly to the deuterated solvent or solvent mixture in the NMR tube.

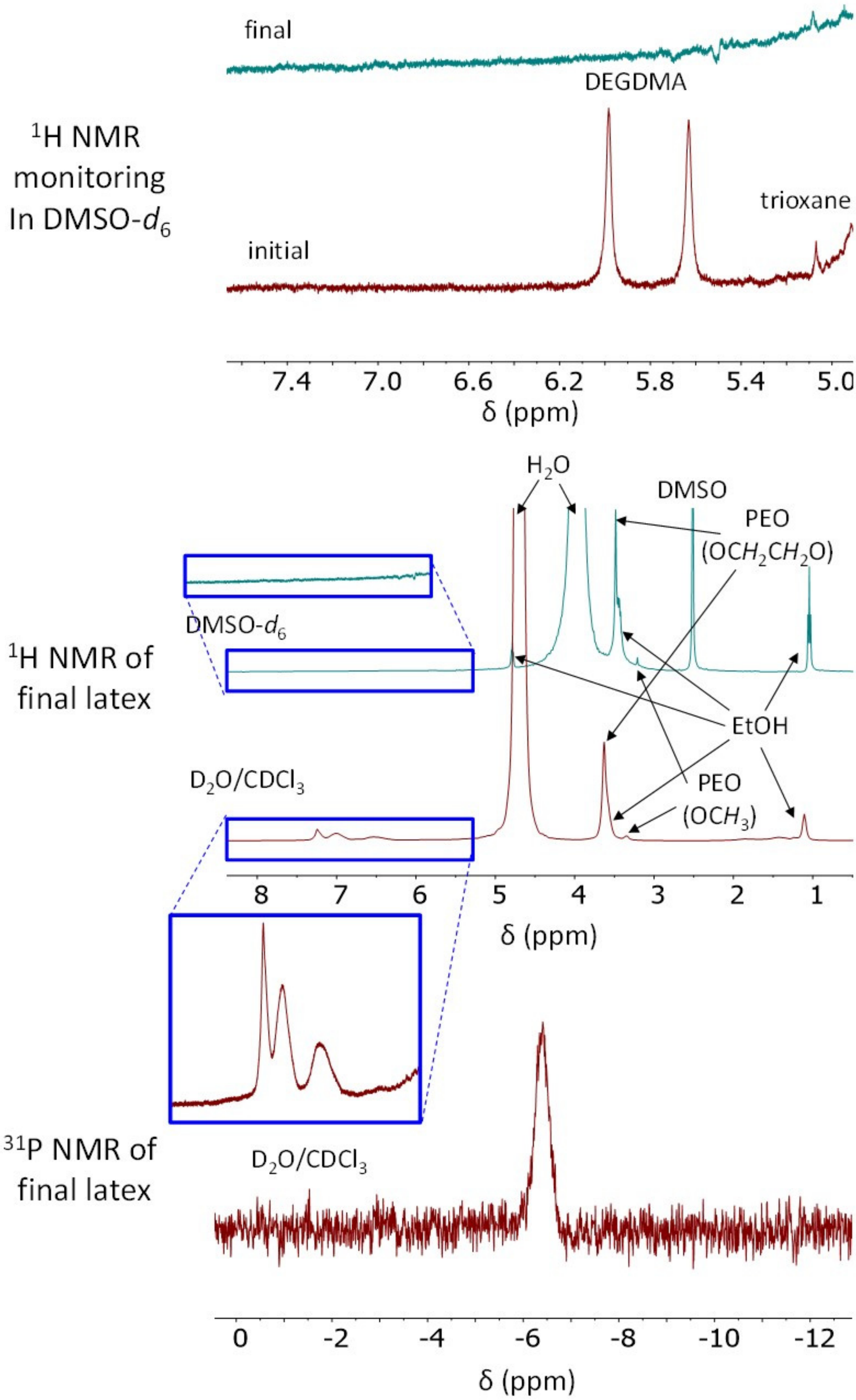

Figure S 32. NMR data for the synthesis of the R_0-[$(SS^-Na^+)_{0.2}$-*co*-$PEOMA_{0.8}$]$_{50}$-*b*-($St_{0.9}$-*co*-$DPPS_{0.1}$)$_{300}$-*b*-$DEGDMA_{90}$-SC(S)S*n*Pr CCM. All NMR samples were prepared by adding a drop of the reaction mixture directly to the deuterated solvent or solvent mixture in the NMR tube.

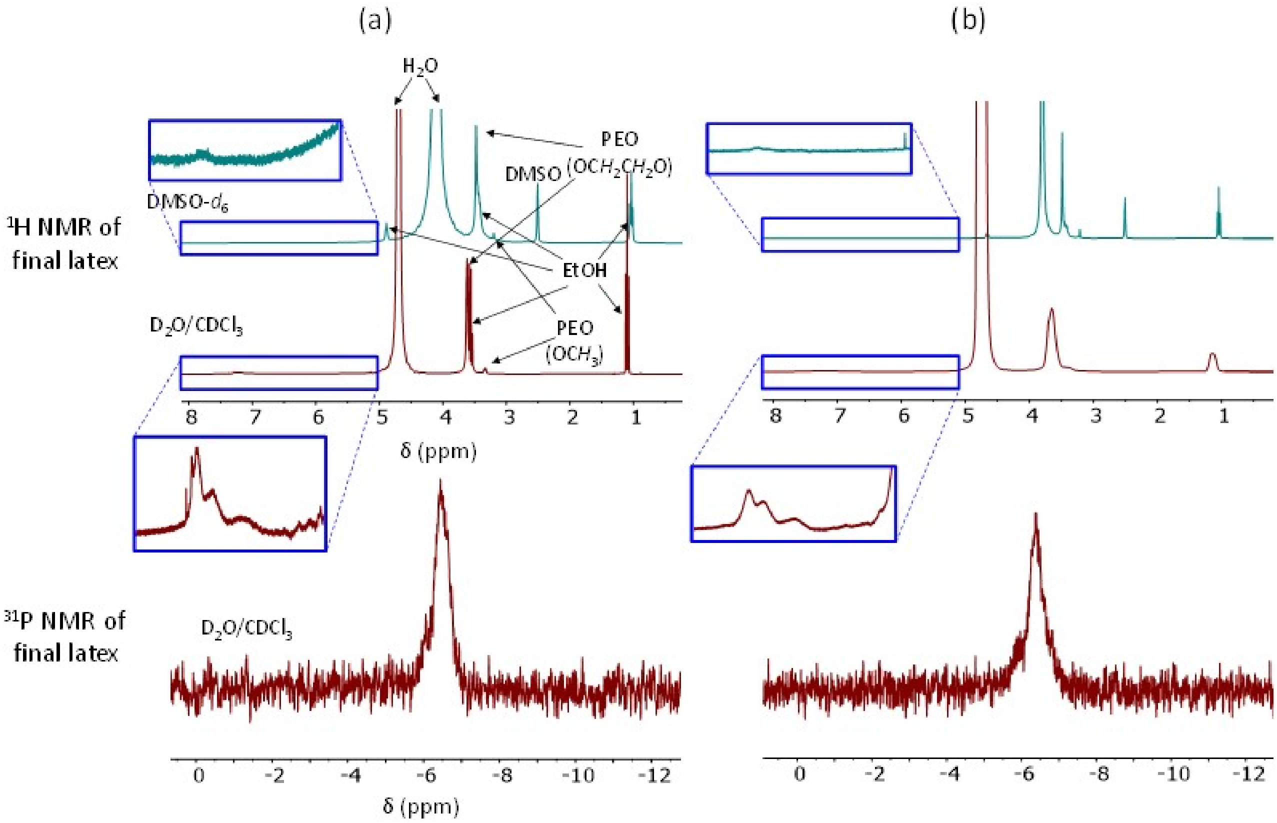

Figure S 33. NMR data for the synthesis of the R$_0$-[(SS$^-$Na$^+$)$_{0.2}$-*co*-PEOMA$_{0.8}$]$_{50}$-*b*-St$_{50}$-*b*-(St$_{425}$-*co*-DPPS$_{30}$-*co*-DEGDMA$_{15}$)-SC(S)S*n*Pr NGs. All NMR samples were prepared by adding a drop of the reaction mixture directly to the deuterated solvent or solvent mixture in the NMR tube.

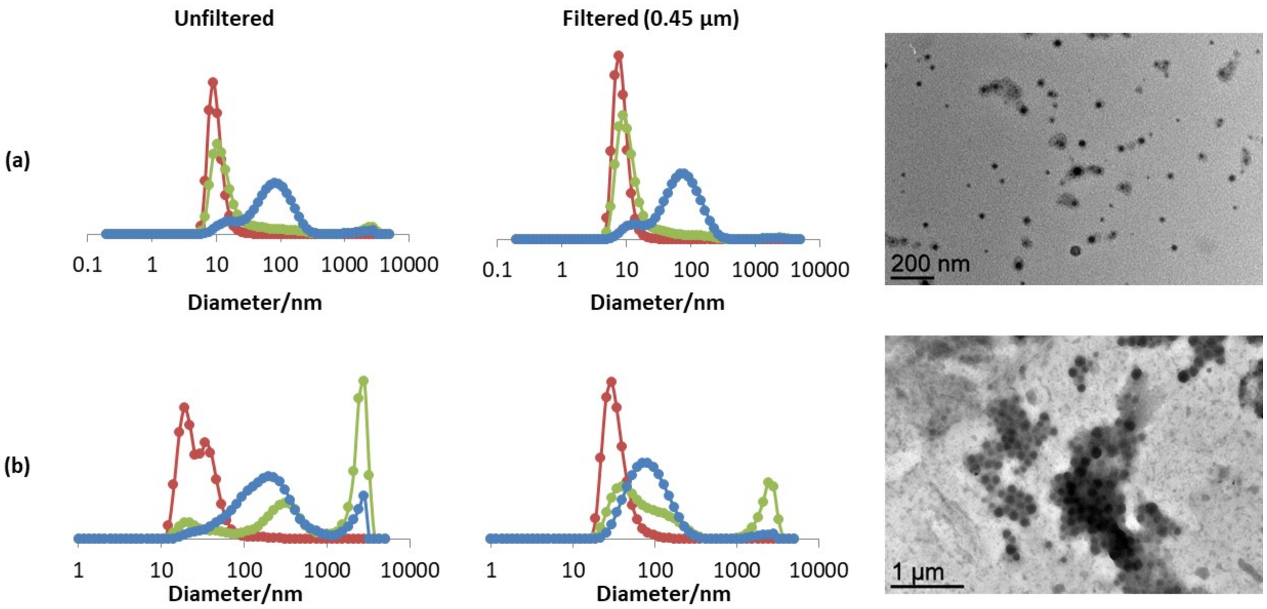

Figure S 34. DLS (left) and TEM (right) characterization of the R$_0$-[(SS$^-$Na$^+$)$_{0.2}$-*co*-PEOMA$_{0.8}$]$_{50}$-*b*-St$_{50}$-*b*-(St$_{425}$-*co*-DPPS$_{30}$-*co*-DEGDMA$_{15}$)-SC(S)S*n*Pr NGs. Color coding for the DLS size distributions: number (red), volume (green) and intensity (blue).

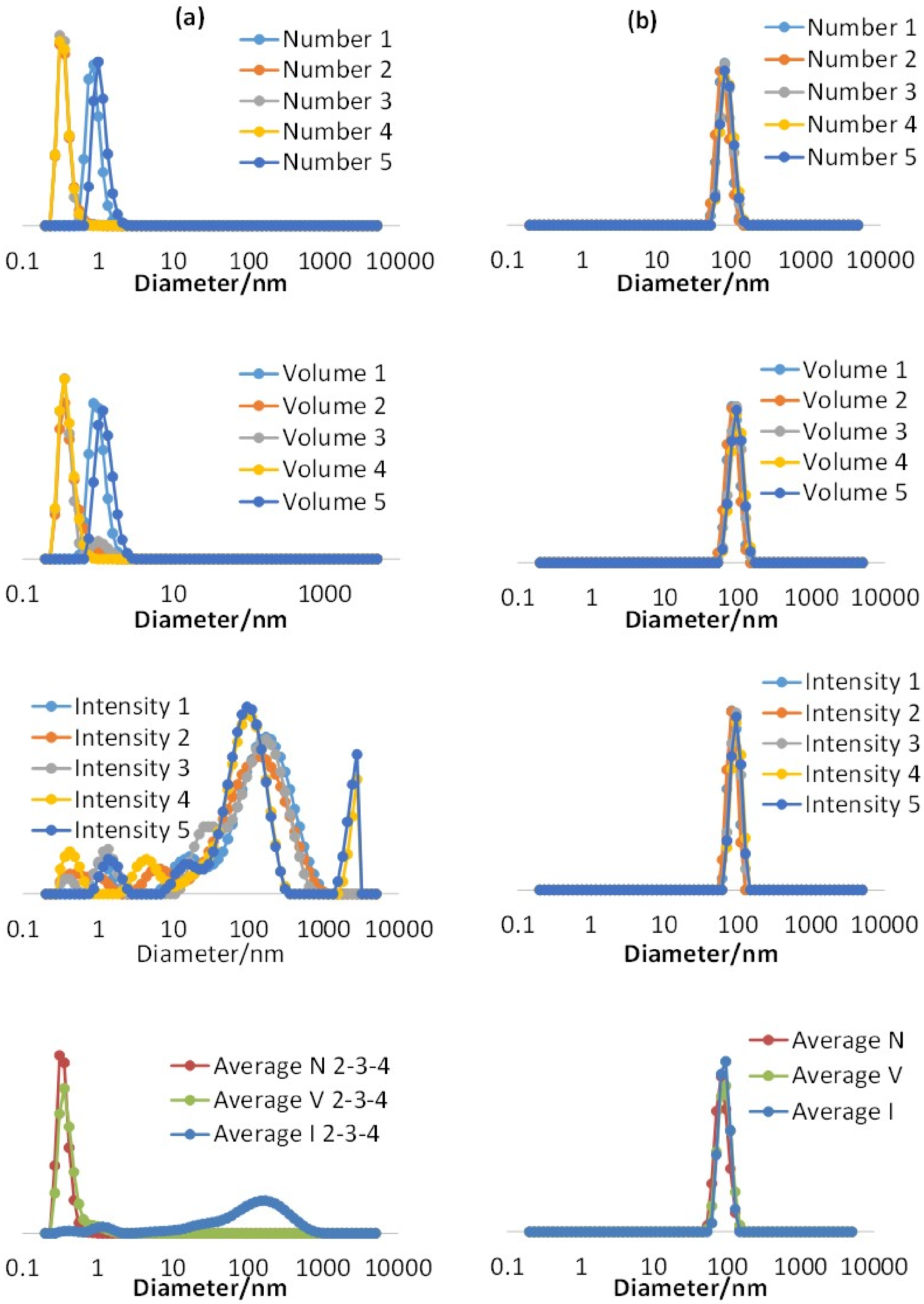

Figure S 35. DLS characterization of (a) the R_0-($SS^{-}Na^{+}_{0.2}$-*co*-$PEOMA_{0.8}$)$_{50}$-*b*-($St_{0.9}$-*co*-$DPPS_{0.1}$)$_{300}$-SC(S)SPr diblock copolymer and (b) the corresponding R_0-($SS^{-}Na^{+}_{0.2}$-*co*-$PEOMA_{0.8}$)$_{50}$-*b*-($St_{0.9}$-*co*-$DPPS_{0.1}$)$_{300}$-*b*-($St_{0.9}$-*co*-$DEGDMA_{0.1}$)$_{150}$-SC(S)SPr CCM in a THF-water 60:40 mixed solvent. The dispersions were filtered through a 0.45 µm pore filter and measurements were done in sequence on the same dispersion every 20 seconds.

Table S 3. Hydrogenation of neat styrene with the [RhCl(COD)(TPP)] precatalyst embedded in CCM nanoreactors.[a]

Additive	Recycle	Conv./%	TON	[Rh]/ppm[b]
R_0-[$(SS^-Na^+)_{0.2}$-*co*-$PEOMA_{0.8}$]$_{140}$-*b*-($St_{0.9}$-*co*-$DPPS_{0.1}$)$_{300}$-*b*-($St_{0.9}$-*co*-$DEGDMA_{0.1}$)$_{150}$-SC(S)S*n*Pr (Table 1, entry 1)	0	29.4	588	6.1
	1	3.52	70	23.2
	2	7.09	142	22.0
	3	6.67	133	26.6
	4	6.12	122	23.7
	5	4.90	98	14.1
	6	8.09	162	13.5
R_0-[$(SS^-Na^+)_{0.2}$-*co*-$PEOMA_{0.8}$]$_{50}$-*b*-($St_{0.9}$-*co*-$DPPS_{0.1}$)$_{300}$-*b*-$DEGDMA_{90}$-SC(S)*SnPr* (Table 1, entry 2)	0	26.3	526	2.4
	1	14.61	292	18.6
	2	4.56	91	1.5
	3	15.06	301	6.3
	4	14.46	289	24.0
	5	12.88	258	7.2
R_0-[$(SS^-Na^+)_{0.2}$-*co*-$PEOMA_{0.8}$]$_{140}$-*b*-St_{50}-*b*-(St_{425}-*co*-$DPPS_{30}$-*co*-$DEGDMA_{15}$)-SC(S)S*n*Pr (Table 1, entry 3)	0	41.9	837	n.d.
	1	8.72	174	
	2	6.72	134	
	3	19.03	381	
	4	3.47	69	
	5	2.67	53	
	6	3.31	66	

[a] Reaction conditions: styrene/Rh = 2000, P/Rh = 4, T = 25°C, p(H_2) = 20 bar, stirring rate = 1200 rpm, reaction time: 2.5 h. [b] From the ICP-MS analysis of the recovered organic phase. n. d. = not determined.

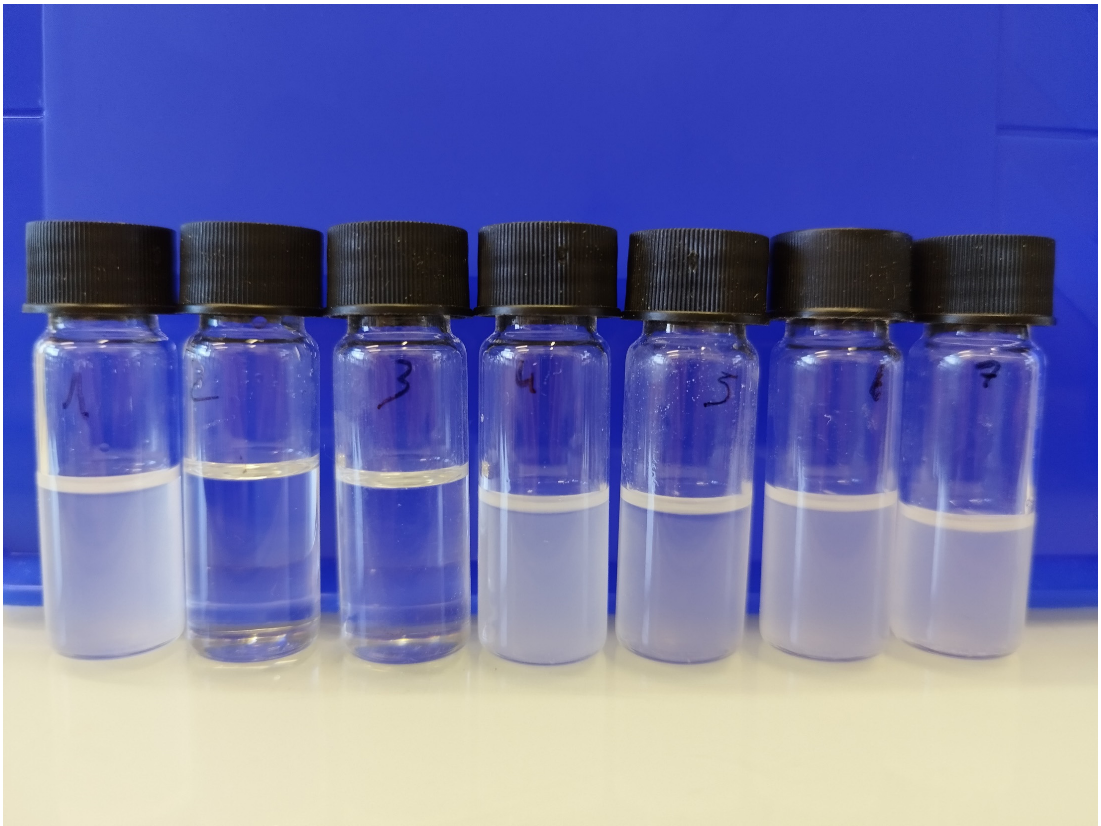

Figure S 36. Recovered reaction mixtures after the recycling runs of Table 1 (entry 1) and Figure 13 (a).

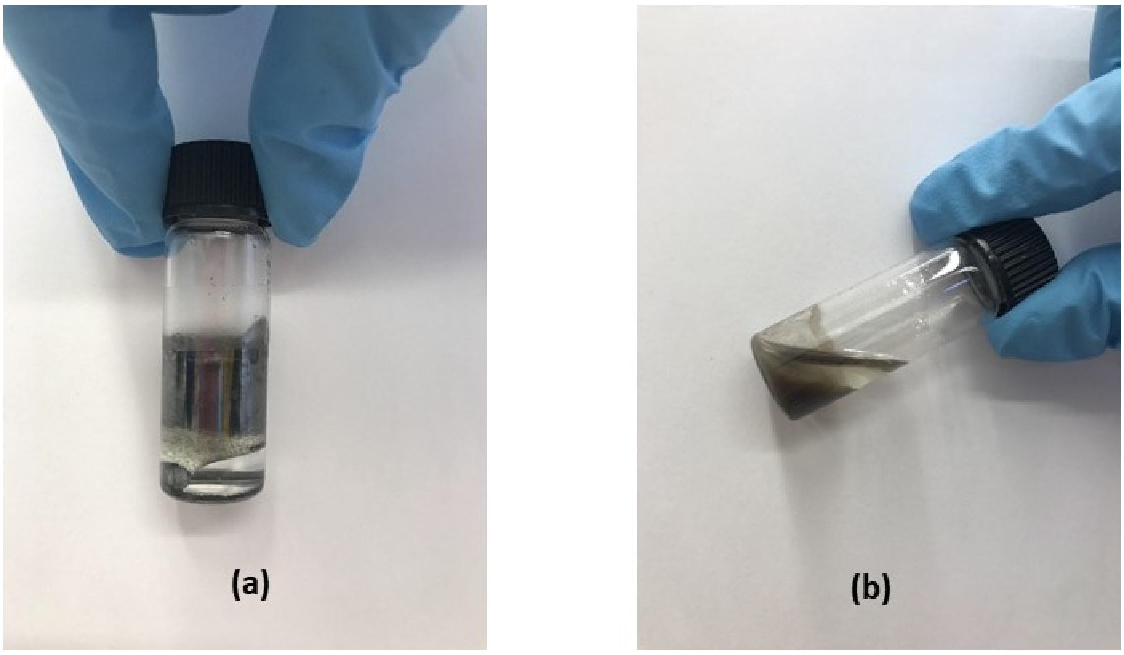

Figure S 37. Recovered reaction mixtures after the catalytic runs of Table 1. (a) Entry 6. (b) Entry 7.

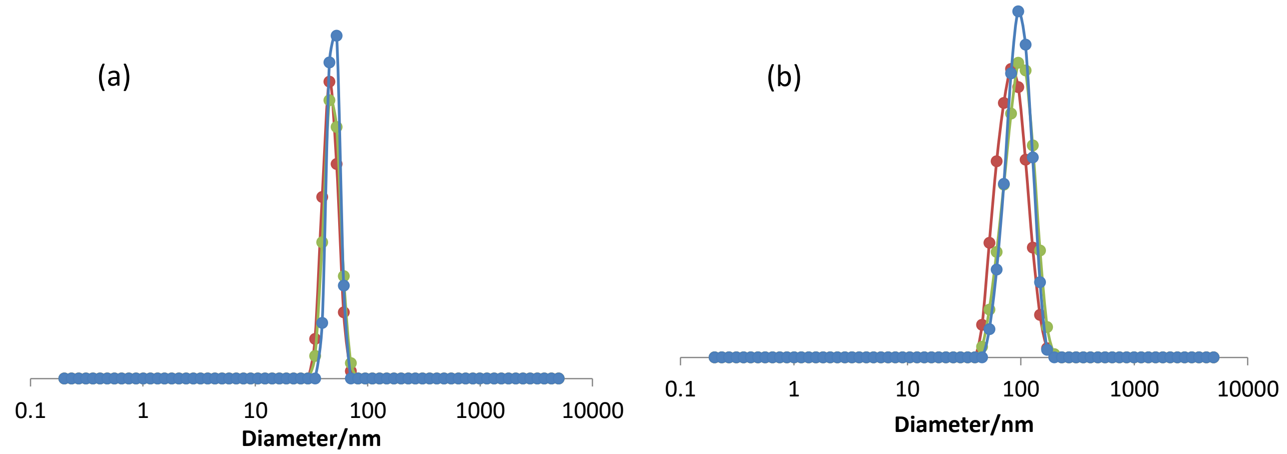

Figure S 38. DLS of the recovered organic phase from the recycling experiments of Table 1, measured after dilution in Et_2O without filtration. (a) First recycle of entry 1. (b) First recycle of entry 2. Color coding for the DLS size distributions: number (red), volume (green) and intensity (blue).
